# RIP3-mediated microglial necroptosis promotes neuroinflammation and neurodegeneration in the early stages of diabetic retinopathy

**DOI:** 10.1038/s41419-023-05660-z

**Published:** 2023-03-29

**Authors:** Zijing Huang, Jiajian Liang, Shaolang Chen, Tsz Kin Ng, Marten E. Brelén, Qingping Liu, Rucui Yang, Biyao Xie, Shuping Ke, Weiqi Chen, Dingguo Huang

**Affiliations:** 1 Joint Shantou International Eye Center of Shantou University and The Chinese University of Hong Kong, Shantou, Guangdong China; 2 Shantou University Medical College, Shantou, Guangdong China; 3 Department of Ophthalmology & Visual Sciences, The Chinese University of Hong Kong, Hong Kong, Hong Kong

**Keywords:** Innate immune cells, Chronic inflammation, Necroptosis

## Abstract

Diabetic retinopathy (DR) is a leading cause of blindness that poses significant public health concerns worldwide. Increasing evidence suggests that neuroinflammation plays a key role in the early stages of DR. Microglia, long-lived immune cells in the central nervous system, can become activated in response to pathological insults and contribute to retinal neuroinflammation. However, the molecular mechanisms of microglial activation during the early stages of DR are not fully understood. In this study, we used in vivo and in vitro assays to investigate the role of microglial activation in the early pathogenesis of DR. We found that activated microglia triggered an inflammatory cascade through a process called necroptosis, a newly discovered pathway of regulated cell death. In the diabetic retina, key components of the necroptotic machinery, including RIP1, RIP3, and MLKL, were highly expressed and mainly localized in activated microglia. Knockdown of RIP3 in DR mice reduced microglial necroptosis and decreased pro-inflammatory cytokines. Additionally, blocking necroptosis with the specific inhibitor GSK-872 improved retinal neuroinflammation and neurodegeneration, as well as visual function in diabetic mice. RIP3-mediated necroptosis was activated and contributed to inflammation in BV2 microglia under hyperglycaemic conditions. Our data demonstrate the importance of microglial necroptosis in retinal neuroinflammation related to diabetes and suggest that targeting necroptosis in microglia may be a promising therapeutic strategy for the early stages of DR.

## Introduction

Diabetic retinopathy (DR) is the most common microvascular complication of diabetes and remains the leading cause of vision loss in working-age adults, resulting in a significant burden on both households and societies [1]. Anti-vascular endothelial growth factor (VEGF) agents and pars plana vitrectomy (PPV) have shown great clinical success in managing the late stages of DR [2, 3]. However, the precise pathogenesis and cellular and molecular events in the early stages of DR, including neuronal degeneration, microvascular abnormalities, and glial cell dysfunction, are not fully understood.

Acute inflammation is a protective mechanism against injuries, while persistent inflammation can worsen the damage and accelerate the progression of the disease. In addition to microvascular changes, laboratory and clinical evidence suggests that inflammation plays a key role and is a therapeutic target in the early stages of DR [4, 5]. Neuroinflammation in the central nervous system is characterized by the activation of glial cells (mainly microglia) and the release of pro-inflammatory cytokines such as tumor necrosis factor-α (TNF-α), interleukin-1β (IL-1β), IL-6, and chemokines such as monocyte chemoattractant protein-1 (MCP-1) [6]. There is a strong correlation between the levels of these pro-inflammatory molecules and the severity of DR [7, 8]. High blood sugar levels can also lead to an influx of leukocytes, called leukostasis, which has been linked to endothelial cell death and disruption of the blood-retinal barrier [9]. However, the precise molecular mechanism of neuroinflammation in the early stages of DR, particularly how neuroinflammation is triggered by glial cells, is not fully understood.

In recent years, a novel form of programmed cell death called necroptosis has gained significant attention due to its role in various pathologies, particularly inflammatory, autoimmune, and neurodegenerative disorders [10–13]. Necroptosis is different from apoptosis, in which cell fragments are engulfed by macrophages without inducing inflammatory responses, and is distinct from necrosis, which is uncontrolled and highly inflammatory [11]. Classic necroptosis is triggered by death receptors such as TNFRs and TLRs, mediated by the activation of the receptor interacting protein kinase 1 (RIP1)/RIP3 complex, and executed by the phosphorylation and oligomerization of mixed lineage kinase domain-like (MLKL) [10, 14]. Targeting cell necroptosis using specific RIP inhibitors has been shown to reduce local inflammation and improve the outcome of various diseases, including inflammatory bowel disease [15], interface dermatitis [16], and donor kidney inflammatory injury after allograft transplantation [17]. Necroptosis has been previously described in retinal detachment [18] and ischemia-reperfusion injury [19], but its role in ischemic retinopathy, specifically the early stages of DR, is not yet clear.

In the present study, using in vivo and in vitro assays, we explore the involvement of necroptosis in microglia and its contribution to neuroinflammation, with the aim of furthering our understanding of DR pathogenesis and potentially providing new therapeutic targets for early DR intervention.

## Materials and methods

### Ethics statement

All animal studies adhered to guidelines for the care and use of laboratory animals issued by the National Institutes of Health (NIH) and were approved by the local Animal Ethic Committee of Joint Shantou International Eye Center of Shantou University and The Chinese University of Hong Kong. Mice were anesthetized with intramuscular injection of zoletil and xylazine. Body temperature was maintained at 37 ± 0.5 °C through a heating pad during surgery. At the end of the experiments, mice were euthanized through carbon dioxide asphyxiation of air.

### Establishment and treatment of streptozotocin (STZ)-induced diabetes model

Streptozotocin (STZ) is an antibiotic that causes pancreatic islet β-cell destruction and is widely used for inducing type 1 diabetes mellitus on experimental models, which are used for evaluating the pathological consequences of diabetes and its complications, and for screening potential therapies [20]. DR phenotypes observed in STZ-induced mice include thinning of retinal layers, loss of retinal cells, vascular hyperpermeability, and increased inflammation and neuroinflammation [21]. C57BL/6 J mice were purchased from Beijing Vital River Laboratory Animal Technology Co., Ltd. C57BL/6-background RIP3-targeted mutant (*rip3*^−/−^) mice were purchased from The Jackson Laboratory. Animals were kept in a specific pathogen-free facility. The streptozotocin (STZ)-induced diabetic retinopathy model was established as previously described [22]. Mice at 8 weeks of age were fasted overnight and then received intraperitoneal injections of STZ (50 mg/kg, Sigma-Aldrich) diluted in 10 mM sodium citrate buffer for 5 consecutive days. Blood glucose levels were measured using the kit with an automatic analyzer (Accu-Chek @Active, Roche) in blood samples from the tail vein. Animals with blood glucose levels maintained at over 16.6 mmol/L (300 mg/dl) were considered as diabetic. Control mice were injected intraperitoneally with sodium citrate buffer. For treatment, the specific RIP3 inhibitor GSK-872 [1 μM resolved in 10% (v/v) DMSO in PBS; Abcam, Cambridge, MA, USA] or vehicle (PBS containing equivalent DMSO) were injected intravitreally on the first day of the 4^th^ week and the first day of the 8^th^ week after STZ induction, respectively. Intravitreal injections were performed using a 5-µL Hamilton syringe with a 33-gauge needle. Twelve weeks after diabetes induction, mice were euthanized and the retinas were collected for further analysis (Supplementary Information 1).

### Immunofluorescence assay

Eyes were removed and fixed in 4% paraformaldehyde (PFA) for 30 min in room temperature. For retinal flat-mounts, retinas were separated carefully from the eyeballs, incubated with primary and secondary antibodies, and then washed in PBST and flat-mounted. For retinal sections, retinas were cryoprotected in Tissue-Tek (Sakura Finetek USA Inc.) at −20 °C overnight and 8 μm cryosections were prepared. Sections were incubated with primary, secondary antibodies, and 4’, 6-diamidino-2-phenylindole (DAPI). The flat mounts and sections were observed using a confocal microscope (Carl Zeiss LSM700, German) or a Leica DM4000 B LED automated upright microscope system. Primary antibodies include anti-iba-1 (Wako Chemicals, 1:100), anti-phospho-MLKL (p-MLKL, 1:100), anti-RIP3, anti-GFAP, anti-TUJ1, anti-TNF-α, anti-Tmem119, anti-brn3a, and anti-CD31 (Abcam, 1:100). The secondary antibodies include donkey anti-rabbit IgG H&L (Alexa Fluor 488, 555), donkey anti-rat IgG H&L (Alexa Fluor 555), and donkey anti-goat IgG H&L (Alexa Fluor 488, 555) antibodies (Abcam, 1:1000). For double staining of p-MLKL and terminal deoxynucleotidyl transferase-mediated dUTP-fluorescein nick end labeling (TUNEL) assay, cryosections were first stained with rabbit anti-p-MLKL and then incubated with donkey anti-rabbit IgG H&L (Alexa Fluor 555) secondary antibody before TUNEL staining (In Situ Cell Death Detection Kit, Fluorescein; Roche, IN, USA) was performed according to the manufacturer’s instructions. For section analysis, 6 retinas from 6 mice in each group were used. Three sections from each retina were randomly chosen and a mean value was determined. For flat-mount assays, 3 images were obtained in different locations of the retina (central, mid-peripheral and peripheral areas) and 6 retinas from 6 mice in each group were used for analysis.

### Quantitative reverse transcription polymerase chain reaction (qRT-PCR)

The mRNA levels of IL-1β, IL-6, TNF-α, and MCP-1 were detected by qRT-PCR. The total RNA of the BV2 cells and retinas were extracted with TRIzol (Invitrogen) and converted into first-strand cDNA using random hexamer primers and the Reverse Transcriptase Superscript II Kit (Invitrogen) according to the manufacturer’s instructions. qRT-PCR was performed in a total volume of 20 μL containing 2 μL of cDNA, 10 μL of 2×SYBR Premix Ex Taq, and 10 μmol/L of the primer pairs. The PCR amplification protocols consisted of 95 °C for 30 s and up to 40 cycles of 95 °C for 5 s and 60 °C for 34 s according to the manufacturer’s instructions. The primers were *il-1β* fwd 5′ TCA GGC AGA TGG TGT CTG TC-3′ and rev 5′- GGT CTA TAT CCT CCA GCT GC-3′; *il-6* fwd 5′-ACT CAC CTC TTC AGA ACG AAT TG-3′ and rev 5′-CCA TCT TTG GAA GGT TCA GGT TG -3′; *tnf-α* fwd 5′-GAG GCC AAG CCC TGG TAT G-3′ and rev 5′-CGG GCC GAT TGA TCT CAG C-3′; *mcp-1* fwd 5′-TAA AAA CCT GGA TCG GAA CCA AA-3′ and rev 5′-GCA TTA GCT TCA GAT TTA CGG GT-3′. *Gapdh* was used as a reference gene.

### Western blotting and enzyme-linked immunosorbent assay (ELISA)

Retina tissue and cells were harvested and lysed in RIPA buffer containing 10 mM Tris-HCl (pH 7.5), 150 mM NaCl, 0.1% sodium dodecyl sulfate (SDS), 1% Nonidet P-40, and 1% sodium deoxycholate, and was supplemented with protease and phosphatase inhibitor mini tablets (Thermo Fisher Scientific, Waltham, MA). The protein concentration was determined with bicinchoninic acid protein assay. Western blotting was performed as previously described [23]. Briefly, 30 μg of protein was loaded per lane on a 10% SDS-PAGE. The membrane blots were saturated with 5% BSA in PBST for 1 h at room temperature and then incubated overnight at 4 °C with antibodies. Primary antibodies used included anti-RIP1 (BD Bio-Sciences, 1:1000), anti-RIP3 (Abcam, 1:1000), anti-phospho-RIP1 (Invitrogen, 1:1000), anti-phospho-RIP3 (Abcam, 1:1000), anti-MLKL (Abcam, 1:1000), anti-phospho-MLKL (Abcam, 1:1000), and anti-β-actin (Abcam, 1:2000) antibodies. Band intensities were measured using the Image J software (US National Institutes of Health). Co-immunoprecipitation assays were performed using the Thermo Scientific Pierce co-IP kit according to the manufacturer’s instruction. The levels of TNF-α and MCP-1 in cultured microglia was detected with ELISA kits (R&D Systems, Minneapolis, MN) according to manufacturer’s instruction.

### Hematoxylin-Eosin (H&E) staining

Eyes were embedded in paraffin and cut into 5μm vertical slices. Retinal sections were washed and stained with hematoxylin buffer for 10 min at room temperature. The sections were rinsed in deionized water and then dipped in 1% Eosin solution for 15 s. After rehydrated in alcohol gradient, slices were washed again and mounted. Histological analyses of retinal tissues were observed under a microscope (Leica DM4000, Germany). Six eyes from 6 mice in each group were analyzed. For each eye, three horizontal sections were randomly chosen and a mean retinal thickness per image was determined. For each section, 4 images were obtained in different locations of the retina (central, mid-central, mid-peripheral and peripheral). Retinal thickness was calculated using the Image-Pro Plus software with a masked protocol.

### Retinal leukostasis

The retinal leukostasis assay was performed according to a previous report [24]. Mice were anesthetized before their chest cavity was opened. A 20-gauge perfusion cannula was inserted into the left heart ventricle. The right atrium was cut with microscissors to create an outflow pathway. Then phosphate buffer saline was perfused to remove erythrocytes and nonadherent leukocytes. Fluorescein-conjugated concanavalin A (Con A, 50 μg/ml dissolved in PBS; Vector Laboratories, CA) was injected to label the adherent leukocytes for 5 min. The unbound lectin was then removed with PBS perfusion. The eyes were enucleated and fixed in 4% paraformaldehyde before the retinas were dissociated and flat-mounted. Leukostasis were observed using a Leica microscope (DM4000, Germany) and total adherent leukocytes per retina were calculated. Six eyes from 6 mice in each group were analyzed.

### Electroretinogram (ERG)

Following 12-h dark adaptation, mice were anesthetized and the pupil was dilated with 0.5% tropicamide eye drops. Two gold plated loop electrodes contacting the corneal surface were used as the active electrodes. A reference electrode was attached to the skin near the eye and a ground electrode was clipped into the skin of the tail. ERG recordings were collected using RETI scan system (Roland Consult, Wiesbaden, Germany) at a sampling rate of 2 kHz. The amplitudes of b-wave were measured from the average of three responses by a set of three flashes of stimulation, using two flash intensities, 3.0 and 10.0 cd s/m^2^. ERG recording was performed on 10 eyes in each group.

### Open field test

The Open field test was carried out using a method similar to a previous report [25]. The apparatus consisted of a cage (45 × 30 × 30 cm) divided into a white open field (300 lux light intensity, 30 × 30 × 30 cm) and a dark zone (no illumination, 15 × 30 × 30 cm) with a door (5 × 5 cm) between them. To test rats in the open field, the mice were placed in the dark chamber for 2 min for adaptation. Thereafter, the door is opened to allow the mice to move freely between the two chambers for 5 min. The time spent in the dark chamber and white open field was video-recorded. Twelve mice in each group were used for statistical analysis.

### Measurement of pupil light reflection

The visual function of experimental animals can be evaluated by measuring pupil constriction in response to a pupillary light reflex [26, 27]. The mice were dark-adapted for 30 min and maintained under anesthesia during examination. The stimulus was provided by a halogen light source (100 W, 12 V) from a surgical microscope (Topcon, Japan). The light source was maintained to focus with 8x objective and aligned perpendicular to the center of the pupil. The stimulus time was 20 s, followed by 60 s of darkness to restore the pupil diameter to baseline level. The pupil status was recorded with the WinFast PVR photography system. The constricted pupil area was measured using the ImageJ software. Twelve eyes from 12 mice in each group were used for statistical analysis.

### Cell culture and treatments

The BV2 murine microglial cell lines were purchased from Kunming Institute of Zoology, Chinese Academy of Sciences, China. The cells were seeded into a 24-well plate with 1 × 10^5^ cells/well, and maintained with Dulbecco’s Modified Eagle’s Medium (DMEM) supplemented with 10% fetal bovine serum (FBS), streptomycin (50 mg/ml, Invitrogen), and penicillin (50 U/L, Invitrogen). For high glucose stimulus, cells were grown in the 50 mM glucose medium (Sigma-Aldrich) for 48 h. For treatments, cells were pre-stimulated with GSK-872 (5 μM) before cultured in high glucose condition for 12 h. D-Mannitol was used as an osmotic control.

### Statistics

All experiments, including western blotting, qRT-PCR, and immunofluorescence assays, were independently repeated 3 times. The number of eyes and animals used in each experiment and the number of images used for analysis were described in specific sections. Data are shown as mean ± standard deviation (S.D). Two-tailed Student’s *t* test was used to detect the differences between two groups while one-way analysis of variance (ANOVA) was carried out to compare multiple groups with post hoc analysis. *P* < 0.05 was considered as statistically significant.

## Results

### Microglial activation and increased production of pro-inflammatory cytokines in early stages of diabetic retinopathy

**Fig. 1:**
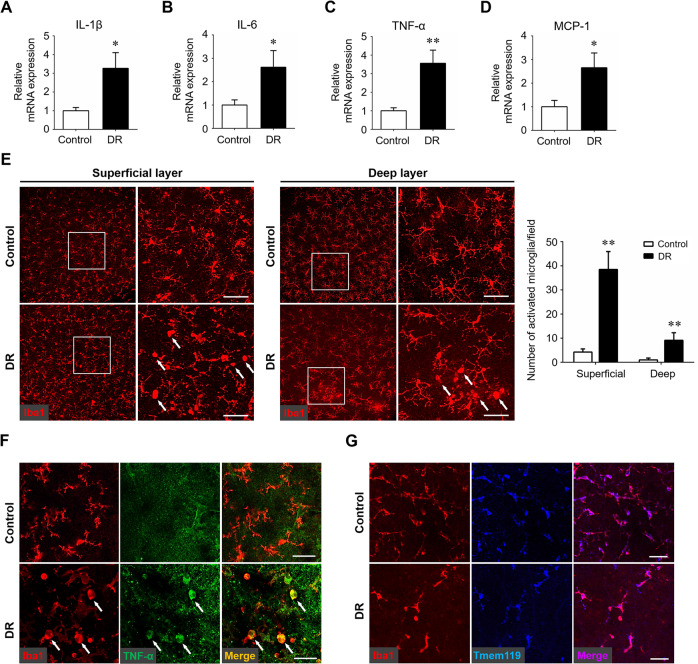
Microglial activation and neuroinflammation cascade in the early stages of diabetic retinopathy. Streptozotocin-induced diabetes model was established and retina samples were collected 12 weeks after diabetes induction. Age-matched non-diabetes mice were used as control. **A**–**D** qRT-PCR array was carried out for several inflammation-related genes, including IL-1β (**A**), IL-6 (**B**), TNF-α (**C**), and MCP-1 (**D**). Data were shown as mean ± S.D. The experiments were repeated 3 times for statistical analysis. **E** Representative images of iba-1 immunostaining on retinal flat-mounts. Arrows indicated activated microglia characterized by swollen cell bodies and short branched processes. Scale bar: 50 μm. Three images were obtained in different locations of the retina (central, mid-peripheral and peripheral areas) and 6 retinas from 6 mice in each group were used for analysis. **F** Immunostaining of iba1 and TNF-α on retinal flat-mounts. Arrows indicated cells co-stained with both iba1 and TNF-α. Scale bar: 50 μm. **G** Immunostaining of iba1 and Tmem119 on retinal flat-mounts. Scale bar: 50 μm. *P < 0.05, **P < 0.01.

Neuroinflammation has been shown to play a critical role in the initiation and development of DR. The inflammatory response in the early stages of DR using the STZ-induced diabetes murine model were evaluated by detecting the levels of key pro-inflammatory cytokines, including IL-1β, IL-6, TNF-α, and MCP-1, which are well-known to facilitate neuroinflammation and exacerbate neural injury in the CNS. The mRNA expression of these cytokines was elevated in the diabetic retinas (Fig. 1A–D). Meanwhile, increased number of activated microglia, characterized by swollen bodies with short processes, was observed in both superficial and deep layers of the diabetic retina, as compared with the resting ones with highly ramified processes in the non-diabetic control (Fig. 1E). In addition, these ameboid-like microglia expressed TNF-α abundantly, suggesting that activated microglia were the main source of pro-inflammatory cytokines (Fig. 1F). The iba1^+^ microglia were distinguished from resident or infiltrating macrophages by immunolabeling of Tmem119, a novel specific microglial marker (Fig. 1G). These results showed that microglia were activated and adopted proinflammatory phenotypes with higher expression levels of TNF-α at early stages of experimental DR.

### Microglia underwent necroptosis in the diabetic retina

**Fig. 2:**
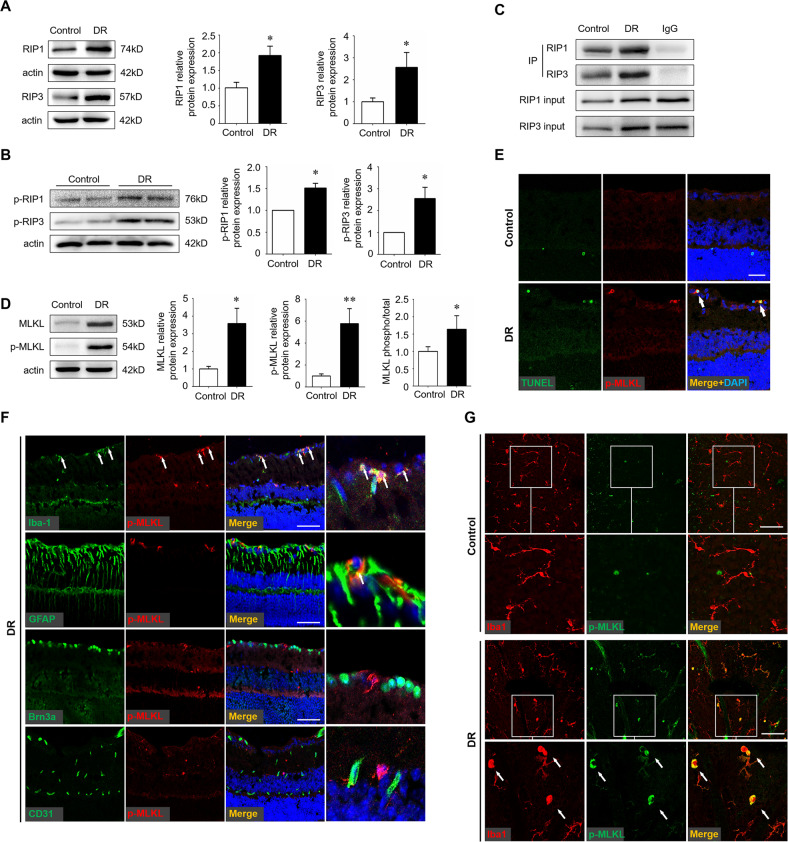
Activation of microglial necroptosis in the diabetic retina. Streptozotocin-induced diabetes model was established and retina samples were collected 12 weeks after diabetes induction. Age-matched non-diabetes mice were used as control. **A**, **B** Protein levels of RIP1 and RIP3 (**A**), and phospho-RIP1 (p-RIP1) and p-RIP3 (**B**), were detected by western blotting. **C** Co-immunoprecipitation assays of RIP1 and RIP3 revealed that RIP1 coimmunoprecipitated with RIP3 in the diabetic retina. **D** Protein levels of MLKL and p-MLKL were detected by western blotting. **E** Combined p-MLKL staining and TUNEL assay on retinal cryosections. Arrows indicated necroptotic cells that were both p-MLKL and TUNEL positive. Scale bar: 50 μm. **F** Double staining of p-MLKL with different cell markers on cryosections of the diabetic retinas. Iba1, a marker for microglia. GFAP, a marker for astrocytes and Müller cells. Brn3a, a marker for retinal ganglion cells. CD31, a marker for vascular endothelial cells. Arrows indicated cells co-stained with p-MLKL and iba1 and partially GFAP. Scale bar: 50 μm. **G** Double staining of p-MLKL and iba1 on flat-mounts of the diabetic retina and non-diabetic control. Arrows indicated cells co-stained with p-MLKL and iba1. Scale bar: 50 μm. Western blotting assays were repeated 3 times. Data were shown as mean ± S.D. *P < 0.05. **P < 0.01.

Since increasing evidence has implicated necroptosis as an active mediator of inflammation, we next investigated whether activated microglia underwent necroptosis in the diabetic retina. The expression of key necroptotic machineries, including RIP1, RIP3, were significantly upregulated (Fig. 2A). Since RIP1 and RIP3 mediate cell death in a kinase activity-dependent manner, we measured phosphorylated RIP1 and RIP3 to support the activation of necroptotic pathway. It was found that both RIP1 and RIP3 were significantly phosphorylated in STZ retina (Fig. 2B). In addition, since RIP3 interacted with RIP1 to form the necrosome, we performed immunoprecipitation assays, which further confirm the presence of necroptosis process (Fig. 2C). Moreover, the level of MLKL and its phosphorylation (p-MLKL), a key executioner of necroptosis, was up to five times higher in the diabetic retinas than non-diabetic controls (Fig. 2D). In addition, we carried out combined p-MLKL immunostaining and TUNEL assays to label retinal cells undergoing necroptosis. The TUNEL^+^ p-MLKL^+^ necroptotic cells were observed in the inner layers of the diabetic retinas instead of non-diabetic controls (Fig. 2E). To further identify the involvement specific cell types in necroptosis, retinal sections were prepared and co-staining of p-MLKL and markers for cells in the inner layer, including microglia, astrocytes, retinal ganglion cells, and vascular endothelial cells, were performed respectively. As shown in Fig. 2F, the key necroptotic machinery p-MLKL was mainly located in iba1^+^ microglia, little expressed in GFAP astrocytes, and was almost negative in other cell types. Co-staining of p-MLKL and iba1^+^ microglia using retinal flat-mounts further confirmed that diabetes-induced necroptosis occurs specifically in microglia (Fig. 2G).

### RIP3 deficiency impaired the activation of microglial necroptosis

**Fig. 3:**
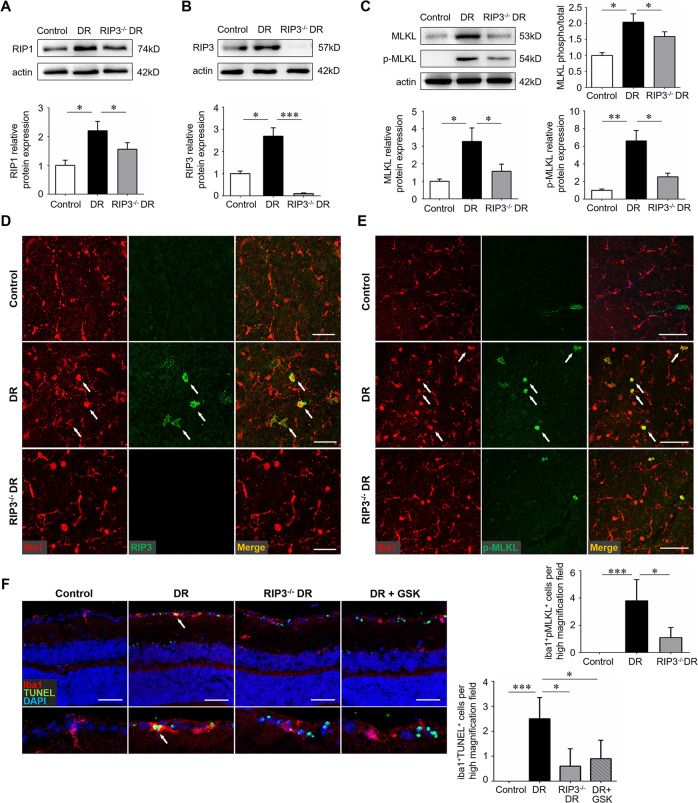
RIP3 deficiency blocked the process of necroptosis in microglia. Wild-type C57BL/6 J and RIP3^−/−^ mice were used to create the streptozotocin-induced diabetes model and retinal samples were collected 12 weeks after diabetes induction. **A**, **B** The protein level of RIP1 and RIP3 was detected by western blotting. **C** The protein level of MLKL and p-MLKL were detected by western blotting. **D** Double immunofluorescence staining of iba1 and RIP3 on retinal flat-mounts. Arrows indicated cells that were both iba1 and RIP3 positive. Scale bar: 50 μm. **E** Double immunofluorescence staining of iba1 and p-MLKL on retinal flat-mounts. Arrows indicated cells that were both iba1 and p-MLKL positive. Scale bar: 100 μm. Three images were obtained in different locations of the retina (central, mid-peripheral and peripheral areas) and 6 retinas from 6 mice in each group were used for analysis. **F** Double immunofluorescence staining of iba1 and p-TUNEL assay on retinal cryosections. Arrows indicated iba1^+^ microglia that were undergoing cell death. Scale bar: 100 μm. Three horizontal sections were randomly chosen and 6 eyes from 6 mice in each group were analyzed. GSK, the specific RIP3 inhibitor GSK-872. Western blotting assays were repeated 3 times. Data were mean ± S.D. *P < 0.05, **P < 0.01, ***P < 0.001.

RIP3 is an essential upstream protein kinase of MLKL and a crucial player in the RIP1-RIP3-MLKL necroptotic signaling pathway [28]. We introduced the RIP3^−/−^ mice to investigate the role of RIP3 in diabetes-induced necroptosis. As expected, knockdown of RIP3 silenced the expression of RIP3 in the diabetic retina while significantly reduced the level of RIP1, MLKL, and p-MLKL, in the diabetic retina (Fig. 3A–C). Consistently, the diabetic retinas of RIP3^−/−^ mice exhibited almost no iba1^+^RIP3^+^ and less iba1^+^p-MLKL^+^ cells as compared to that of normal mice (Fig. 3D, E). In addition, both RIP3 gene knockdown and RIP3 inhibition using a specific inhibitor GSK872 led to a decrease in microglia cell death (Fig. 3F), further indicating that RIP3 deficiency could block the RIP1/RIP3/MLKL necroptotic pathway in DR.

### Blockade of microglial necroptosis rescued diabetes-mediated retinal neuroinflammation and neurodegeneration

**Fig. 4:**
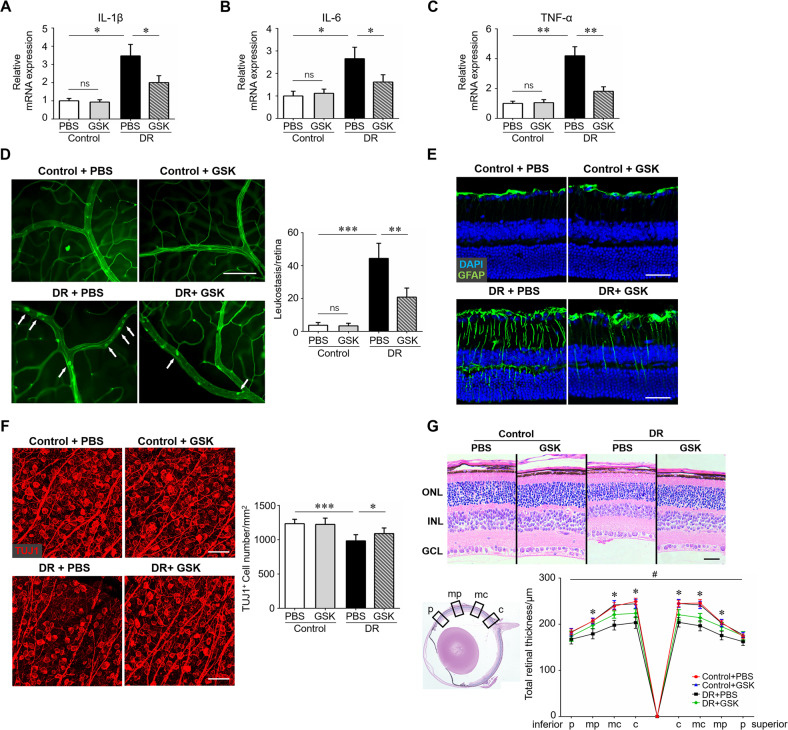
RIP3 inhibition attenuated retinal neuroinflammation and neurodegeneration. Streptozotocin-induced diabetes model was established. Age-matched non-diabetes mice were used as control. GSK-872 or vehicle were injected intravitreally on the first day of the 4th week and 8th week after STZ induction. Twelve weeks after diabetes induction, retina samples were collected and RNA was isolated using TRIzol Reagent. **A**–**C** qRT-PCR array was carried out for inflammation-related genes, including IL-1β, IL-6, and TNF-α. **D** Retinal leukostasis was performed using fluorescein-conjugated concanavalin A to label the adherent leukocytes. The number of adherent leukocytes were observed using a Leica microscope. Total adherent leukocytes per retina and six retinas from 6 mice in each group were used for analysis. Arrows indicated adherent leukocytes. Scale bar: 100 μm. **E** Immunostaining of GFAP, a marker for astrocytes and Müller cells, on retinal cryosections. Scale bar: 100 μm. **F** Immunostaining of TUJ1, a marker for neurons, on retinal flat-mounts. Scale bar: 50 μm. Three images were obtained in different locations of the retina (central, mid-peripheral and peripheral areas) and 6 retinas from 6 mice in each group were used for analysis. **G** H&E staining was carried out to evaluate retinal thickness in each group. Scale bar: 100 μm. Retinal thickness at different locations, including central (c), mid-central (mc), mid-peripheral (mp), and peripheral (p), were analyzed. Three horizontal sections were randomly chosen and 6 eyes from 6 mice in each group were analyzed. *DR + PBS vs DR + GSK, *P* < 0.05. GSK, the specific RIP3 inhibitor GSK-872. ONL outer nuclear layer, INL inner nuclear layer, GCL ganglion cell layer. Western blotting assays were repeated 3 times for statistical analysis. Data were shown as mean ± S.D. *P < 0.05, **P < 0.01. ***P < 0.001, ns no significance.

We next explored whether blocking necroptosis in microglia using a specific RIP3 inhibitor could attenuate retinal neuroinflammation and neurodegeneration in the diabetic retina. We first assessed the binding of several RIP3 inhibitors by molecular docking assays, through which GSK-872 was considered an optimal candidate for RIP3 inhibitors (Supplementary information 2). As shown in Fig. 4A–C, inhibition of RIP3 and the necroptotic signaling pathway using GSK-872 significantly down-regulated the mRNA levels of several key pro-inflammatory cytokines, including IL-1β, IL-6, and TNF-α. Leukostasis and gliosis, two major events in neuroinflammation and early pathologic changes in DR, was evaluated. The leukostasis assay revealed severe leukocytes adhesion in the diabetic micro-vessels, which was largely ablated by GSK-872 treatment (Fig. 4D). Gliosis was evaluated using GFAP immunostaining. In the normal retina, GFAP was mainly localized to astrocytes in the superficial layer. In the diabetic retina, intensive GFAP labeling in astrocytes and Müller cells in the inner layer was detected, whereas RIP3 inhibition using GSK-872 significantly reduced GFAP immunoreactivity as compared to the controls (Fig. 4E). Retinal neurodegeneration is an early pathology of DR that can precede visible vasculopathy [29]. Of note, diabetes induced a significant decrease in both neuron density and neuro-retinal thickness, which can be partially rescued by GSK-872 treatment (Fig. 4F, G). It was also noted that necroptosis inhibition by GSK-872 administration had no visible impact on non-diabetic mice.

### Necroptosis inhibition with GSK-872 improved visual function of the diabetic mice

**Fig. 5:**
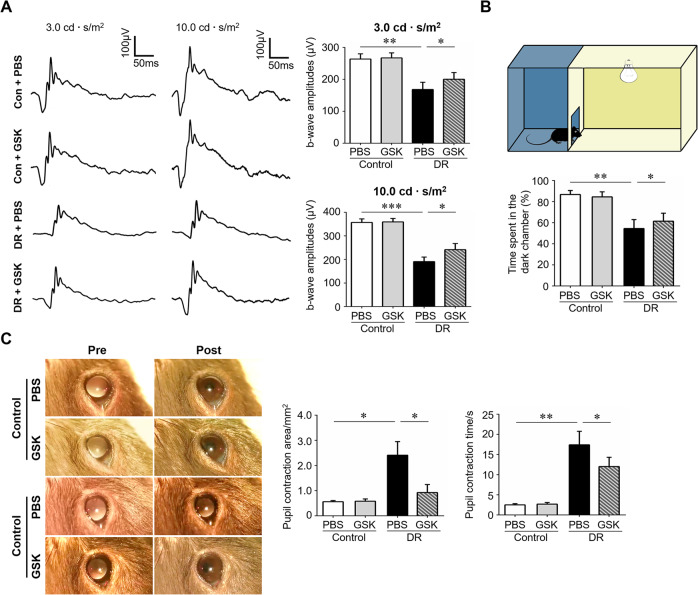
RIP3 inhibition improved visual functions and light sensitivity of the diabetic mice. Streptozotocin-induced diabetes model was established. Age-matched non-diabetes mice were used as control. GSK-872 or vehicle were injected intravitreally on the first day of the 4th week and 8th week after STZ induction. The experiments were carried out 12 weeks after diabetes induction. **A** Electroretinogram (ERG) was performed using two flash intensities, 3.0 cd·s/m^2^ and 10.0 cd·s/m^2^. The amplitudes of b-wave were measured from the average of three responses by a set of three flashes of stimulation. *n* = 10 eyes in each group. **B** The open field test was carried out to test the light sensitivity and visual functions of the mice. The device consisted of a cage (45 × 30 × 30 cm) divided into a white open field and a dark zone with a door (5 × 5 cm) between them. The time spent in the dark chamber was recorded. *n* = 12 mice in each group. **C** Pupillary light reflex measured by pupil constriction was conducted to evaluate RGC function. *n* = 10 eyes in each group. GSK, the specific RIP3 inhibitor GSK872. Data were presented as mean ± S.D. *P < 0.05, **P < 0.01, ***P < 0.001.

To explore whether blocking of microglial necroptosis and neuroinflammation could enhance the light sensitivity and visual function of diabetic mice, ERG was first carried out to measure the electrical potential change of the entire retina in response to light stimulation. The results showed significant improvements of b-wave amplitudes at different light intensities (3.0 and 10.0 cd·s/m^2^) after dark-adapted condition, which represented the neural signals derived from inner-layer neurons, in the GSK-872-treated diabetic mice (Fig. 5A). In addition, an open field test was introduced to assess the light sensitivity and visual functions of the mice based on the theory that normal mice avoid open and bright spaces while those with retinal diseases spend a decreased amount of time in the dark area [30, 31]. This innate tendency can thus be utilized as the basis of a simple test of their ability to light detection. The test revealed that diabetic mice spent less time in the dark chamber whereas GSK-872 treatment partially abolished this effect, implicating increased light sensitivity of the GSK-872-treated mice (Fig. 5B). We also evaluated the pupillary light reflex by measuring the pupil constriction in the diabetic mice with and without treatment. As a result, diabetic mice presented a larger pupil area with light stimulation and longer pupil contraction time than non-diabetic ones. GSK-872 treatment greatly improved the pupillary light reflex by enhancing the contraction amplitude while decreasing contraction time (Fig. 5C). Collectively, these data demonstrated that blockade of microglial necroptosis with GSK-872 improved the light sensitivity and visual function of diabetic mice.

### RIP3-mediated necroptosis was activated in BV2 microglia under glycemic condition

**Fig. 6:**
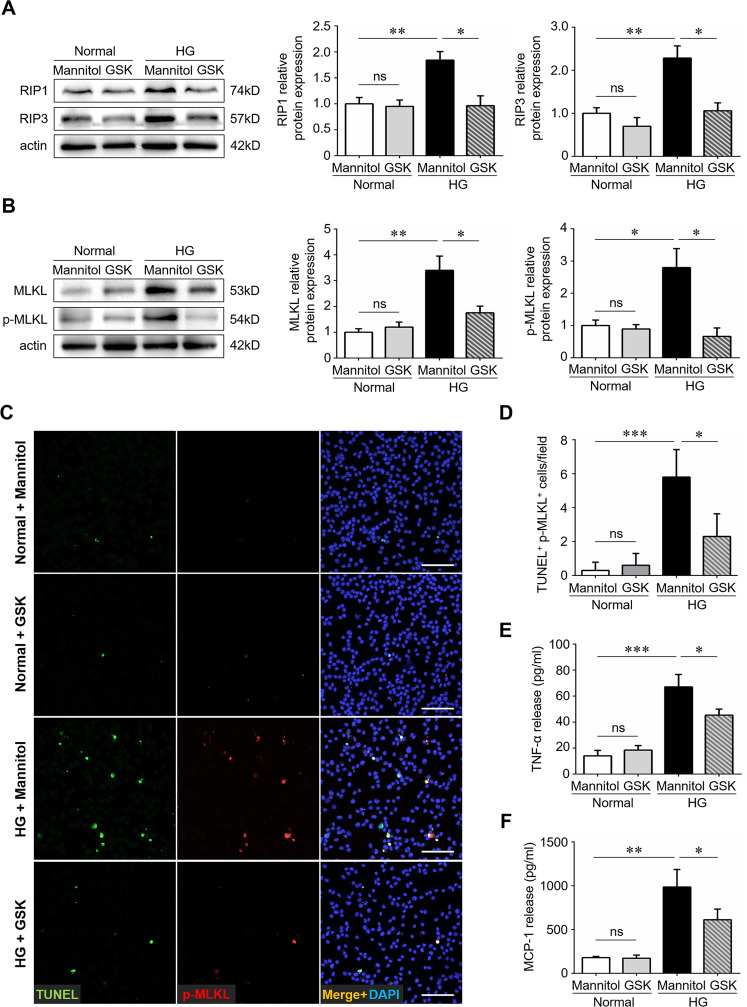
High glucose triggered necroptosis in BV2 microglia. The BV2 murine microglial cell lines were treated with high glucose (50 mM) for 48 h. For treatments, cells were pre-stimulated with GSK-872 (5 μM) before high glucose stimulation for 12 h. D-Mannitol was used as an osmotic control. **A** The protein levels of RIP1 and RIP3 were detected by western blotting. **B** The protein levels of MLKL and p-MLKL were detected by western blotting. **C**, **D** Combined p-MLKL immunostaining and TUNEL assays on BV2 microglial cells under high glucose and normal condition. Ten images in each group were randomly chosen for analysis. **E**, **F** The levels of TNF-α and MCP-1 in cultured microglia was detected using TNF-α and MCP-1 ELISA kit respectively. HG high glucose. GSK, the specific RIP3 inhibitor GSK-872. Western blotting and ELISA assays were repeated 3 times. Data were mean ± S.D. *P < 0.05, **P < 0.01, ***P < 0.001, ns no significance.

We finally investigated whether microglia underwent necroptosis and contributed to the inflammatory cascade in vitro. BV2 microglial cell lines were treated with high glucose or isosmotic mannitol controls. GSK-872 was added before high glucose stimulation. Consistent with the in vivo studies, short-term high glucose exposure induced overexpression of necroptotic machineries, including RIP1, RIP3, MLKL, and p-MLKL, in cultured microglial cells, which could be ablated by GSK-872 treatment (Fig. 6A, B). Co-immunostaining assays revealed the presence of p-MLKL^+^TUNEL^+^ cells under glycemic condition, indicating that microglia cells were undergoing necroptosis. Similarly, GSK-872 supplement rescued microglial cells from necroptotic death (Fig. 6C, D). In addition, RIP3 inhibition by GSK-872 significantly down-regulated the levels of TNF-α and MCP-1 in high glucose-stimulated microglial cells (Fig. 6E, F), suggesting that blocking the necroptotic signaling could orchestrate high glucose-mediated inflammation cascade in vitro.

## Discussion

Although diabetic retinopathy (DR) is primarily a retinal microvascular disease, increasing evidence now shows that various molecular abnormalities characteristic of inflammation are present in the retinas of diabetic patients and animals, as well as in retinal cells exposed to high blood sugar levels, linking inflammatory processes to the development of DR [4, 32]. Diabetes can cause various inflammatory changes in the retina, including upregulation of NF-κB signaling and other pro-inflammatory transcription factors [33], glial activation with elevated levels of pro-inflammatory cytokines and chemokines [34, 35], leukostasis and platelet aggregation [36], increased vascular permeability [37], and others [38]. Microglial activation, a hallmark of neuroinflammation, has been identified as playing an emerging role in DR pathology.

Microglia are resident immune cells in the brain and retina that serve as immune surveillance and provide host defense. When they encounter danger signals, microglia become activated to reduce nociceptive stimulus and maintain tissue homeostasis. However, persistent stress can cause microglia to become over-activated, resulting in the production of large amounts of pro-inflammatory cytokines and chemokines, which can worsen neuronal damage [39, 40]. The precise molecular mechanism of microglia-based neuroinflammation is not fully understood. In recent years, the concept of microglia polarization has been proposed, in which activated microglia are classified into pro-inflammatory “M1” and anti-inflammatory “M2” phenotypes based on specific cytokines and surface antigens [41]. Targeting microglia polarization has been suggested as an alternative strategy for preventing and treating retinal neurodegeneration [42] and ischemic retinopathy [43]. However, the M1/M2 terminology may limit research on the characterization of microglia activation as microglia can assume a diversity of phenotypes and shift functions under different circumstances [44, 45]. It is important to explore novel and specific mechanisms of microglial activation and neuroinflammation.

Necroptosis, a novel form of regulated cell death, has been observed in different cell types in diseased retinas, including photoreceptors in retinal detachment [18], neuronal cells in retinal ischemia-reperfusion injury [19], and retinal pigment epithelium in age-related macular degeneration [46]. We have previously demonstrated the role of microglial necroptosis in retinal degeneration and neuroinflammation, focusing on TLR signaling and the specific inhibition of RIP1 by necrostatin-1 [47]. In this study, we identified the role of microglial necroptosis in the early stages of DR. Importantly, the necroptotic microglia mainly assumed a classically activated “ameboid” shape, indicating that activated microglia are more prone to undergo necroptosis. We also found that necroptosis in microglia contributed to the neuroinflammatory pathogenesis in the diabetic retina, as evidenced by increased production of key pro-inflammatory cytokines such as TNF-α, IL-1β, and MCP-1. Blocking necroptosis with a specific RIP3 receptor successfully downregulated the levels of these inflammatory mediators, further supporting the hypothesis that microglial necroptosis has pro-inflammatory and detrimental effects. Our findings may broaden the scope for microglia-mediated neuroinflammation and necroptosis-targeting therapies for the early stages of DR.

Retinal neurodegeneration has been increasingly recognized as an early event in the pathogenesis of DR that precedes and contributes to microvasculopathy [48]. In this study, blocking microglial necroptosis significantly prevented the reduction of neuronal cell density and neuroretinal thickness in the diabetic retina, which are histological markers of retinal neurodegeneration [49], suggesting that necroptotic microglia may be a therapeutic target for neuroprotection. In addition, an open field test was used to evaluate the visual function following anti-necroptosis treatment, based on the natural aversion of normal mice to open and bright areas [50] and the observation that mice with retinal neurodegeneration spend less time in dark spaces [25]. In this study, mice treated with GSK-872 spent more time in the dark area compared to untreated diabetic mice, indicating that blocking necroptosis was able to attenuate retinal neurodegeneration or restore neuron function. Finally, GSK-872 treatment partially rescued the electrophysiological response to flashes of light and suppressed the impairment of the pupillary light reflex, further supporting the neuroprotective effect of inhibiting microglial necroptosis in diabetic mice.

Necrostatin-1 (Nec-1), a specific RIP1 kinase inhibitor, has been used to block the necroptotic RIP1/RIP3/MLKL pathway and has been shown to be effective in several neurological diseases [51, 52]. However, recent studies have revealed non-death-related biological functions of RIP1 beyond necroptosis, such as directly activating NF-κB signaling and promoting cell survival [53, 54]. In this study, we used GSK-872 to inhibit the activity of RIP3, which is a core and specific molecular machinery for necroptosis [28]. Intravitreal injection of GSK-872 significantly downregulated the level of MLKL, a major necroptosis effector, and suppressed TUNEL staining on microglia in the diabetic retina, indicating that GSK-872 is a promising candidate compound for anti-necroptotic strategies.

In this study, we noticed a modest activation of p-MLKL even in the absence of RIP3. Indeed, it has been described that RIP3-independent modifications on the MLKL kinase facilitate necroptosis execution, including the activation of membrane-associated TAM kinases [55] and other unknown kinases [56]. In addition, phosphorylation and translocation of MLKL to the autophagosome membrane also contribute to cell autophagy via a RIP3-independent pathway [57]. These findings may explain the RIP3-independent partial activation of p-MLKL in our study. Further study using MLKL gene knockout strategies might help to verify this hypothesis.

There are several limitations to this study. First, we evaluated the role of necroptosis using a STZ-induced murine model, which mimics the early stages of DR pathology. However, patients with DR often suffer from severe visual loss when diabetic edema or neovascular complications occur, and VEGF plays a well-recognized role in increased permeability and retinal neovascularization [58]. The effect of necroptosis inhibition on neovascularization, as well as the neuroprotective potential of necroptosis-targeting therapy on late stages of DR, requires further investigation. In addition, the necroptotic machinery p-MLKL in the diabetic retina was slightly expressed on GFAP + astroglia (astrocytes and Müller cells), which also participate in the process of neuroinflammation. Tmem119 was used to differentiate resident microglia from circulating myeloid cells, but the contribution of peripheral myeloid cells to the current disease process cannot be completely ruled out. Further study is needed to confirm the potential role of necroptosis in these cell types.

Although the primary insult in DR may not be inflammation, the immune cell-mediated neuroinflammatory cascade can exacerbate the progression of microvascular injuries and neurodegeneration. Our data demonstrate the presence of microglial necroptosis in the early pathogenesis of DR, which amplifies neuroinflammatory processes and exacerbates neuronal damage in the diabetic retina. Targeting the necroptosis process in microglia may provide a promising therapeutic alternative for early stages of DR.

## Supplementary information

Co-author approves for adding a new author

aj-checklist

Supplementary information 1. Establishment and treatment of STZ-induced diabetic retinopathy mice.

Supplementary information 2. Molecular docking assays.

Supplementary information 3. Original western blot gels

## Data Availability

The data that support the findings of this study are available from the corresponding author upon reasonable request.
