# Supplementary material for: RIP3-mediated microglial necroptosis promotes neuroinflammation and neurodegeneration in the early stages of diabetic retinopathy
Source: Cell Death Dis. 2023 Mar 29;14(3):227. doi: 10.1038/s41419-023-05660-z (PMC10060420; doi:10.1038/s41419-023-05660-z)
Supplement: Supplementary file 3 — Supplementary information 1. Establishment and treatment of STZ-induced diabetic retinopathy mice. [file 41419_2023_5660_MOESM3_ESM.docx]

**
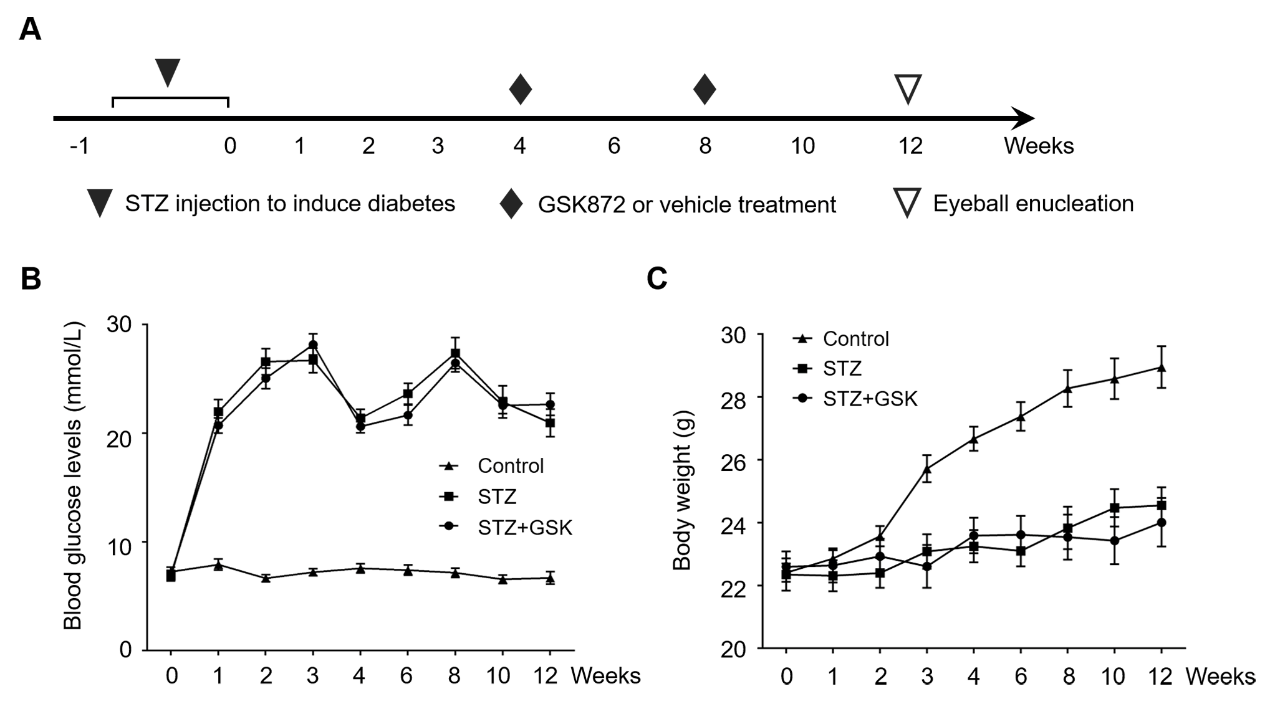
**

**Supplementary information 1. Establishment and treatment of STZ-induced diabetic retinopathy mice.** (**A**) A diagram showing the establishment of STZ-induced diabetes model and treatment of GSK-872 or vehicle. (**B**) Blood glucose levels of STZ-induced diabetic mice treated with or without GSK-872 and non-diabetic control. (**C**) Body weights of STZ-induced diabetic mice treated with or without GSK-872 and non-diabetic control. GSK-872, a specific inhibitor for RIP3.
