# Supplementary material for: RIP3-mediated microglial necroptosis promotes neuroinflammation and neurodegeneration in the early stages of diabetic retinopathy
Source: Cell Death Dis. 2023 Mar 29;14(3):227. doi: 10.1038/s41419-023-05660-z (PMC10060420; doi:10.1038/s41419-023-05660-z)
Supplement: Supplementary file 4 — Supplementary information 2. Molecular docking assays. [file 41419_2023_5660_MOESM4_ESM.docx]

**Supplementary information 2. Molecular docking assays.** The 3D structures of target small molecule ligand compounds GSK-872, GSK-843, GSK-840, and HS-1371, were downloaded from Pubchem database (<https://pubchem.ncbi.nlm.nih.gov/>). The PDB file of the target protein RIP3 (PDB ID: 4m67) was downloaded from the PDB database (<https://www.rcsb.org/>). AutoDock4 and AutoDockTools 4 were used to dehydrate, hydrogenate and charge RIP3 and select torsion bonds for the 4 small molecules and export them into PDBQT format files. Molecular docking was performed using Lamarckian GA mode, and Binding energy, Ligand Efficiency, Total energy, Hydrogenbonds formed and other docking results were used to evaluate the binding ability of the ligands and receptor. Finally, we used PyMOL Molecular Graphics System (<http://www.pymol.org>) to beautify the 3D interaction structure of the docked complex and to export images. 2D interaction images were derived by uploading target protein and ligand structure files to the online website (<https://proteins.plus/>).


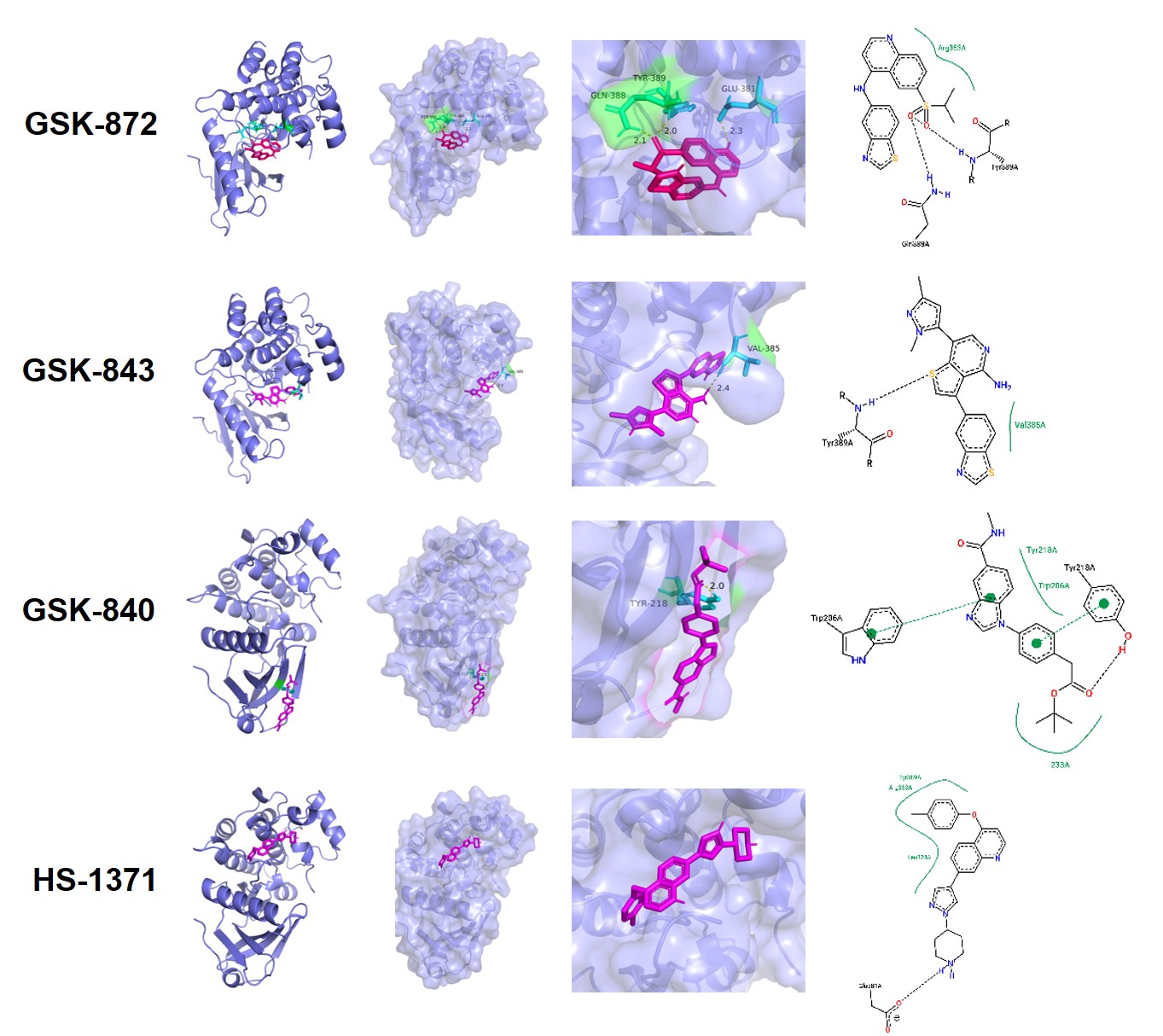


| **Parameters** | **GSK-872** | **GSK-843** | **GSK-840** | **HS-1371** |
| --- | --- | --- | --- | --- |
| Binding energy | -5.22 | -5.57 | -5.62 | -6.14 |
| Ligand efficiency | -0.2 | -0.21 | -0.21 | -0.21 |
| Inhib constant (μM) | 148.28 | 82.6 | 76.43 | 31.36 |
| Intermol energy | -6.12 | -6.17 | -7.41 | -7.34 |
| vdw hb desolv energy | -6.06 | -6.09 | -7.39 | -7.2 |
| Electrostatic energy | -0.06 | -0.08 | -0.02 | -0.13 |
| Total energy | -0.43 | -0.33 | -0.93 | -1.03 |
| Torional energy | 0.89 | 0.6 | 1.79 | 1.19 |
| Unbound energy | -0.43 | -0.33 | -0.93 | -1.03 |
| Hydrogen bonds formed | 2 | 0 | 0 | 0 |
