# Supplementary material for: RIP3-mediated microglial necroptosis promotes neuroinflammation and neurodegeneration in the early stages of diabetic retinopathy
Source: Cell Death Dis. 2023 Mar 29;14(3):227. doi: 10.1038/s41419-023-05660-z (PMC10060420; doi:10.1038/s41419-023-05660-z)
Supplement: Supplementary file 5 — Supplementary information 3. Original western blot gels [file 41419_2023_5660_MOESM5_ESM.pdf]

Western blot gels

Figure 2

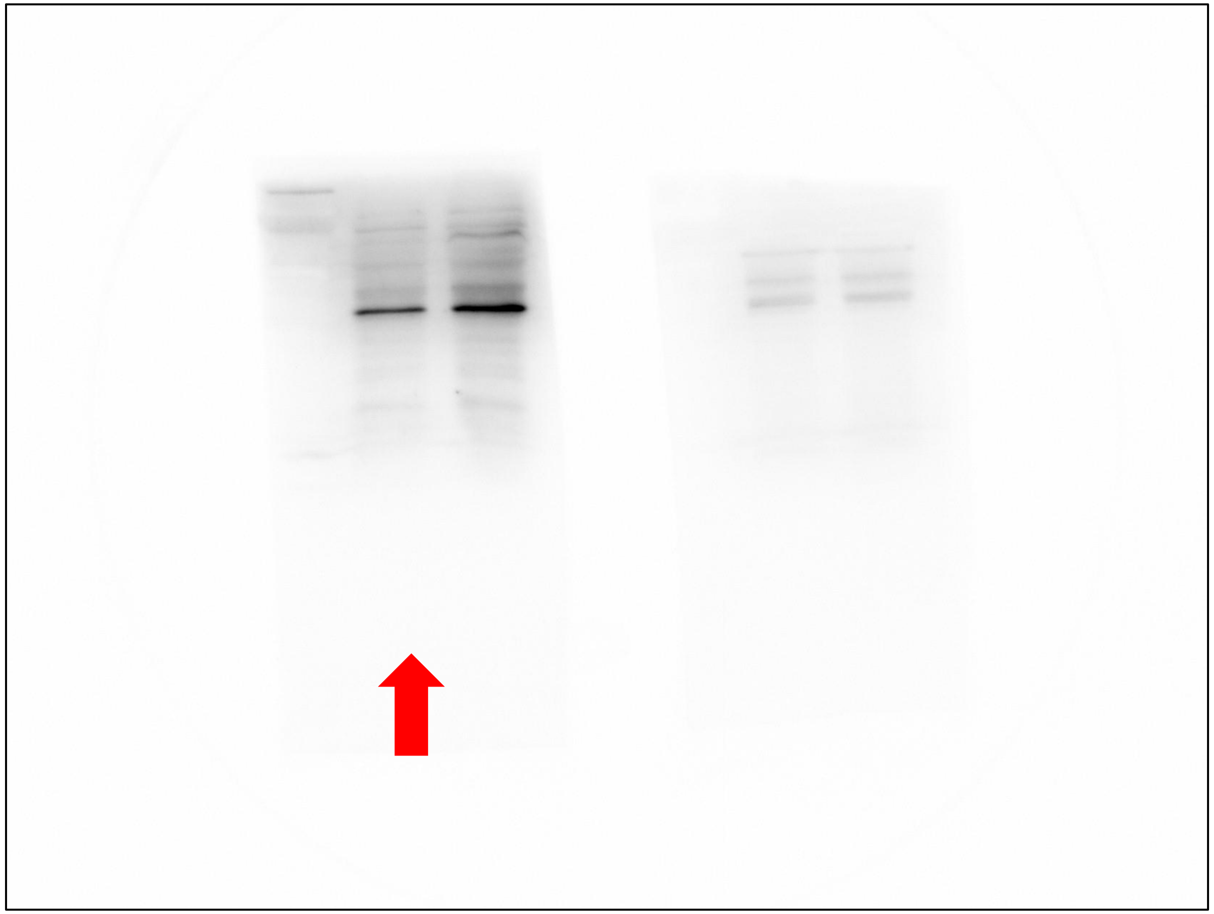

WB_Fig2_RIP1

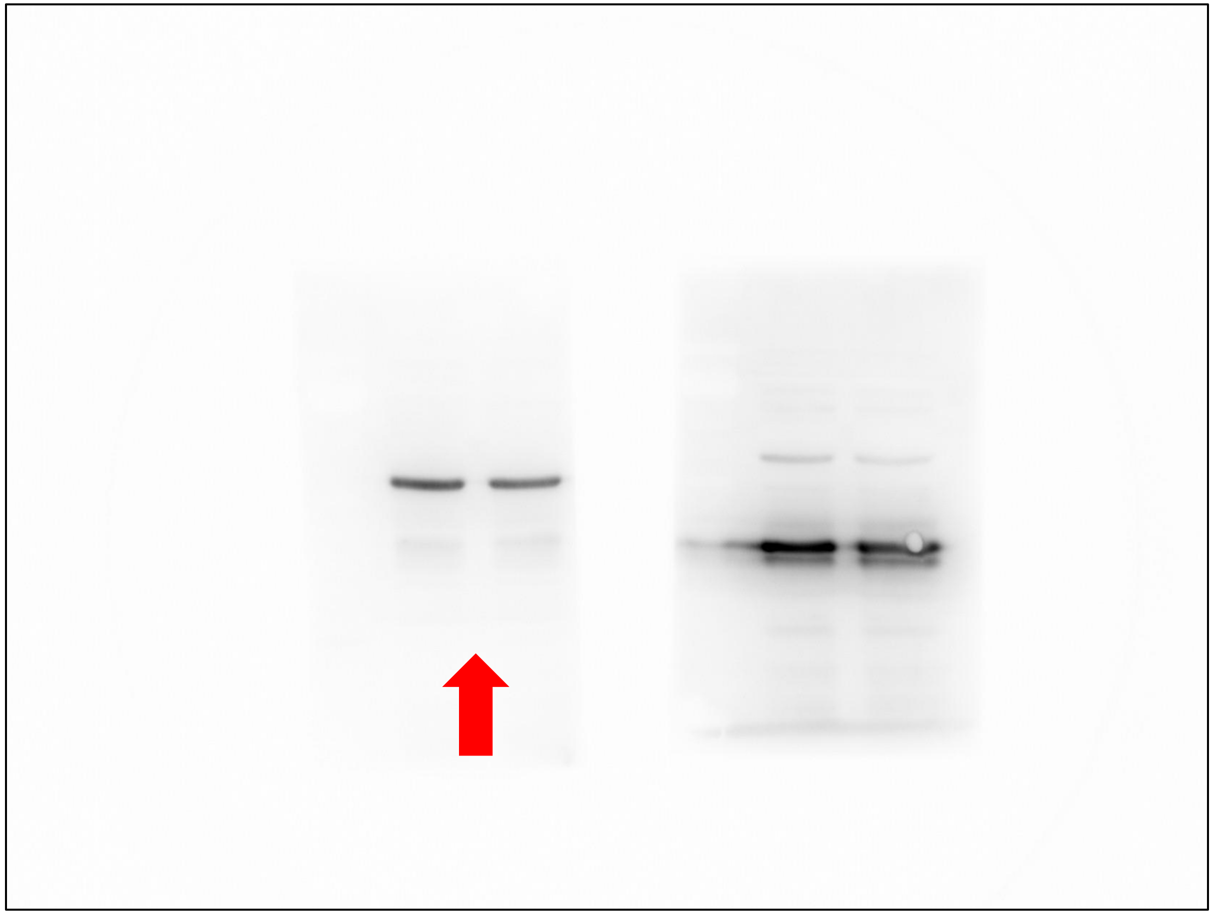

WB_Fig2_RIP1_ACTIN

Figure 2

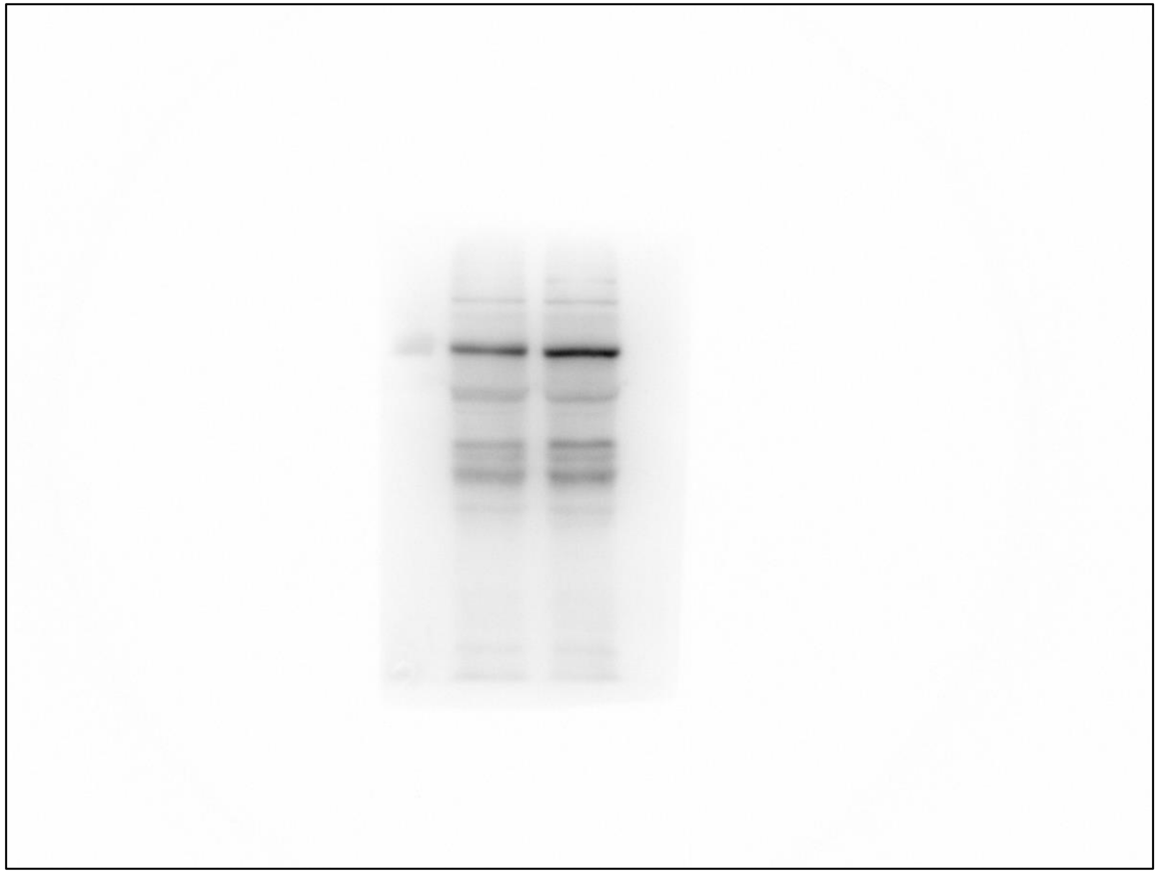

WB_Fig2_RIP3

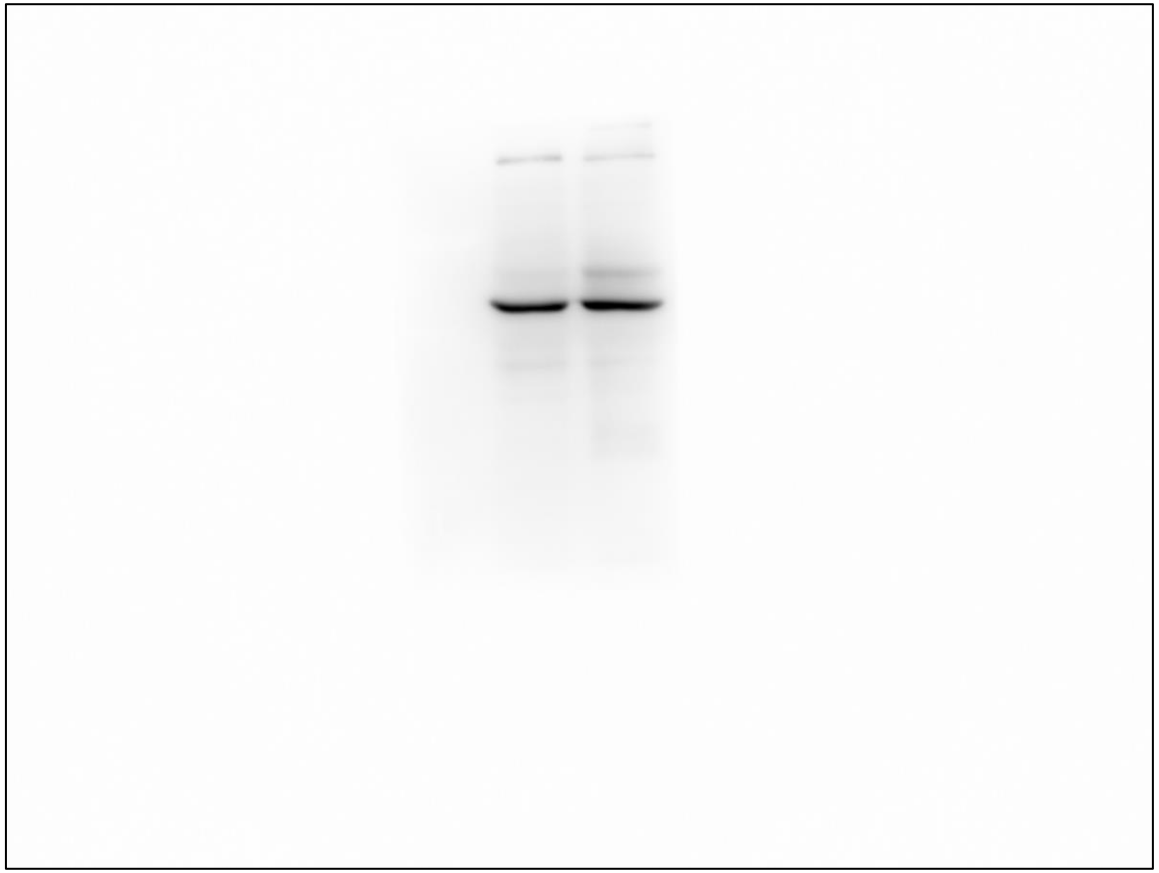

WB_Fig2_RIP3_ACTIN

Figure 2

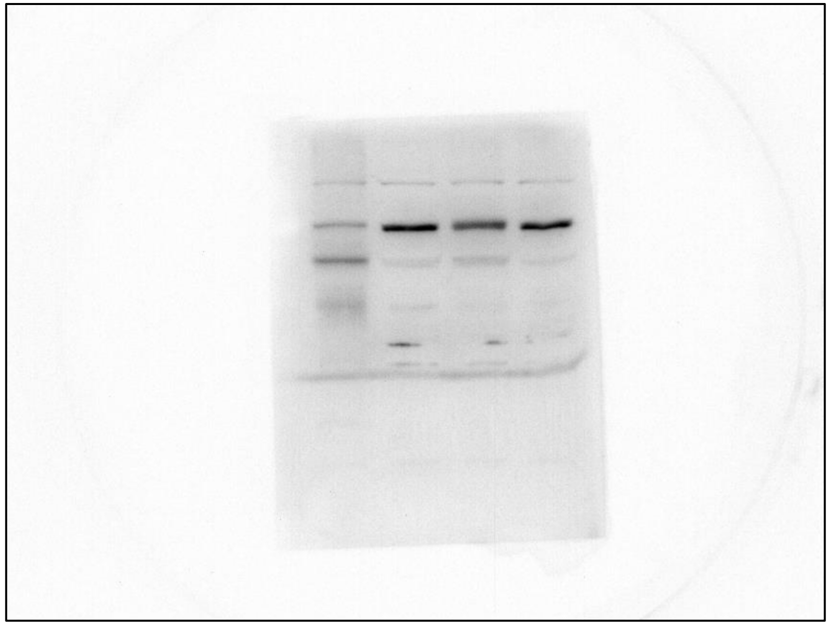

WB_Fig2_MLKL

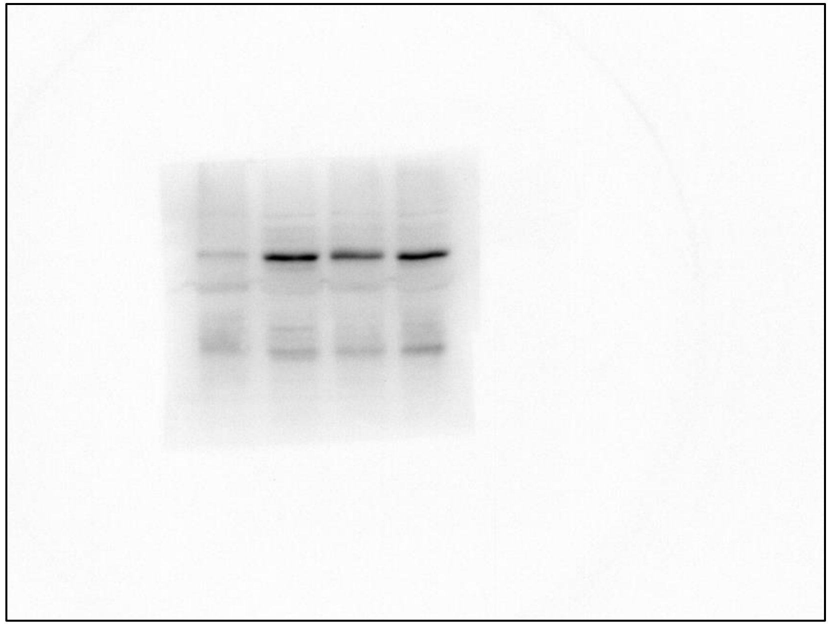

WB_Fig2_pMLKL

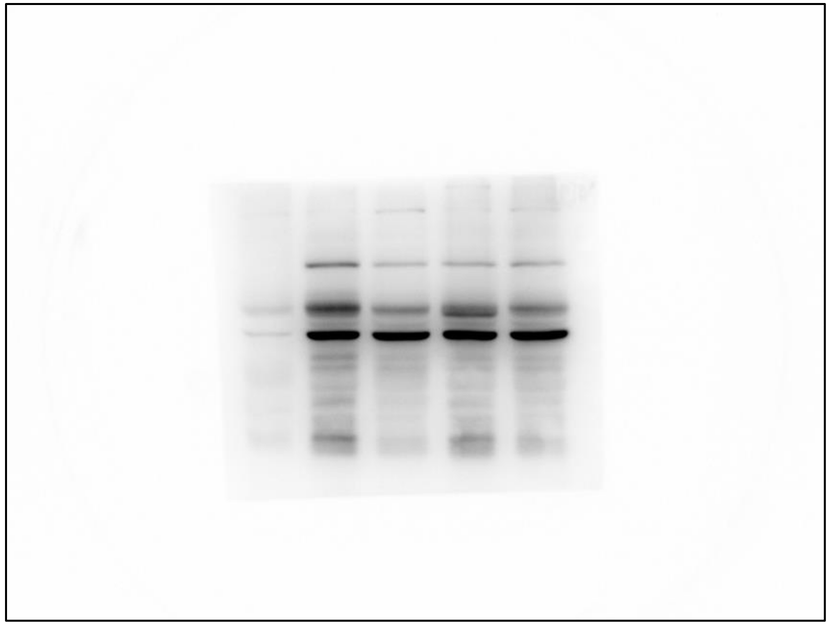

WB_Fig2_(p)MLKL_actin

Figure 3

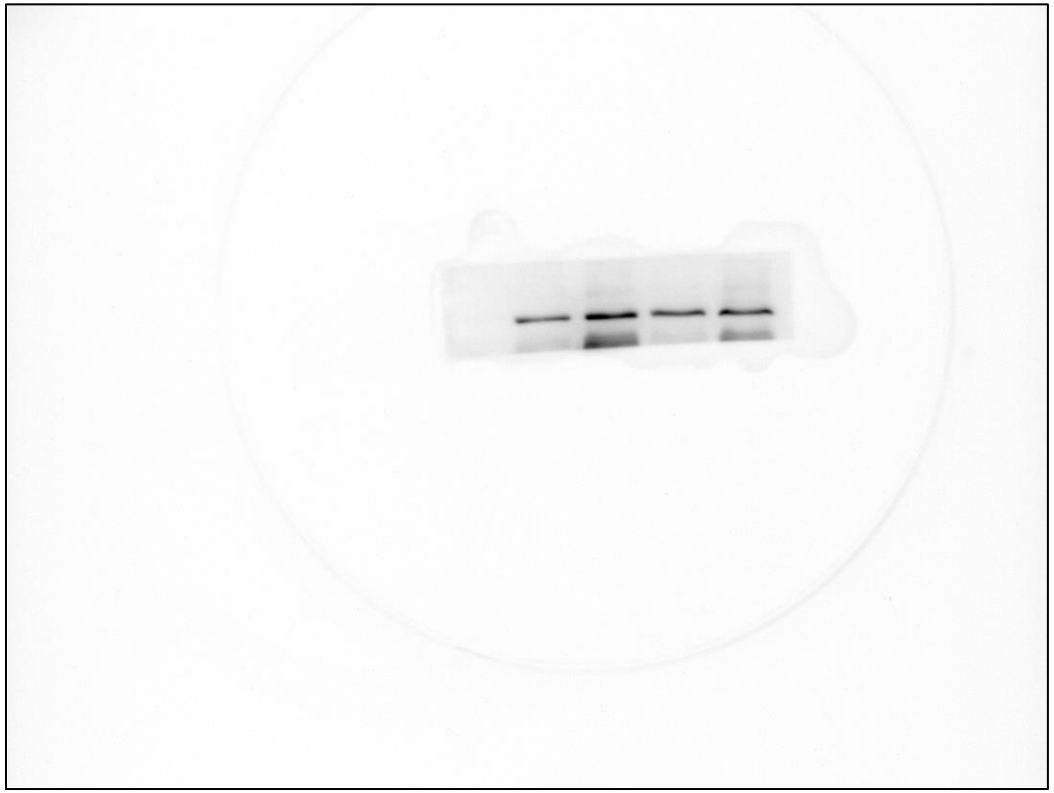

WB_Fig3_RIP1

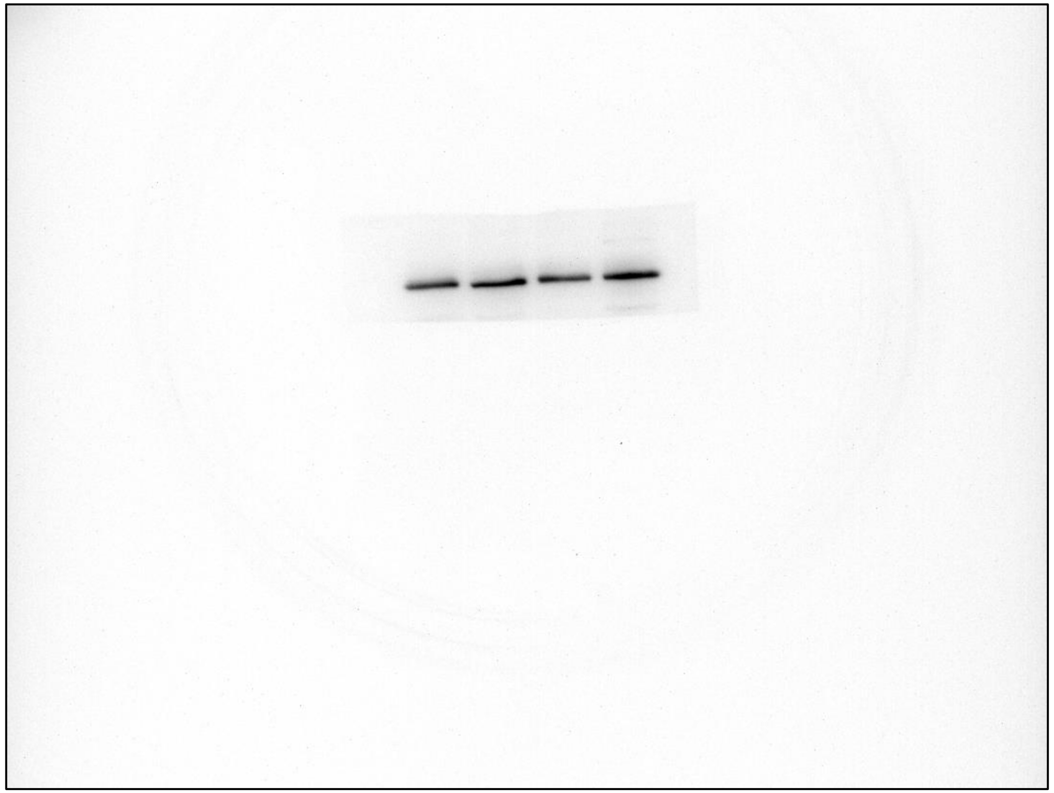

WB_Fig3_RIP1_actin

Figure 3

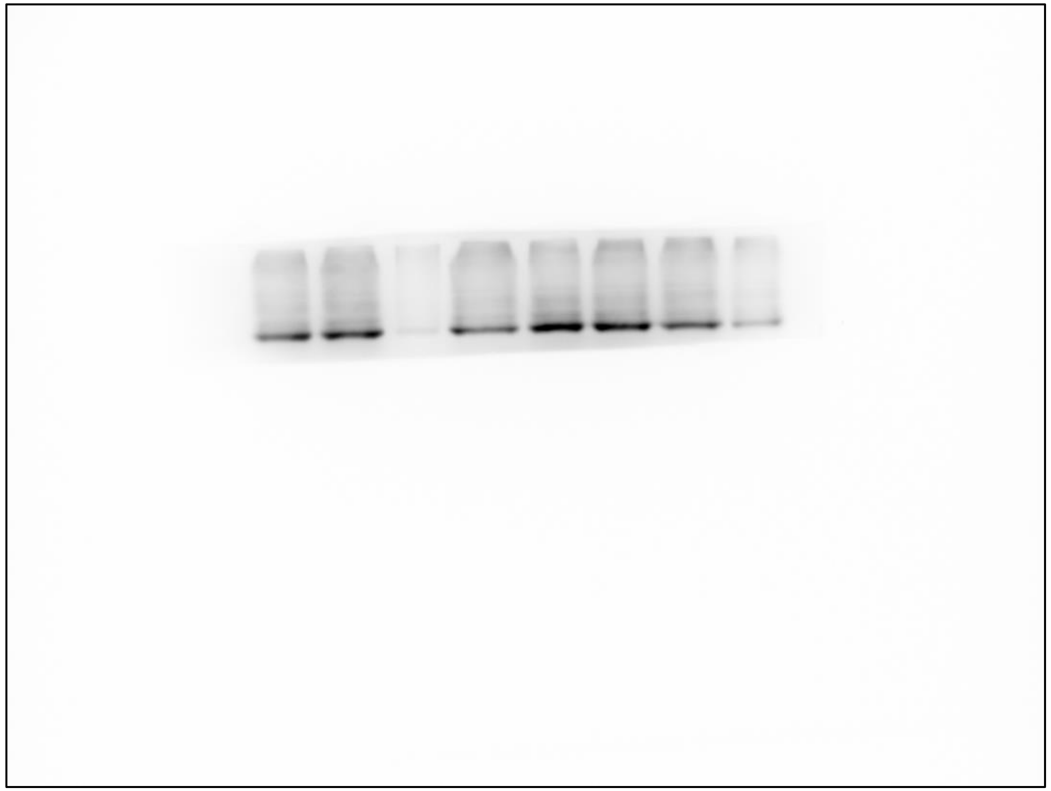

WB_Fig3_RIP3

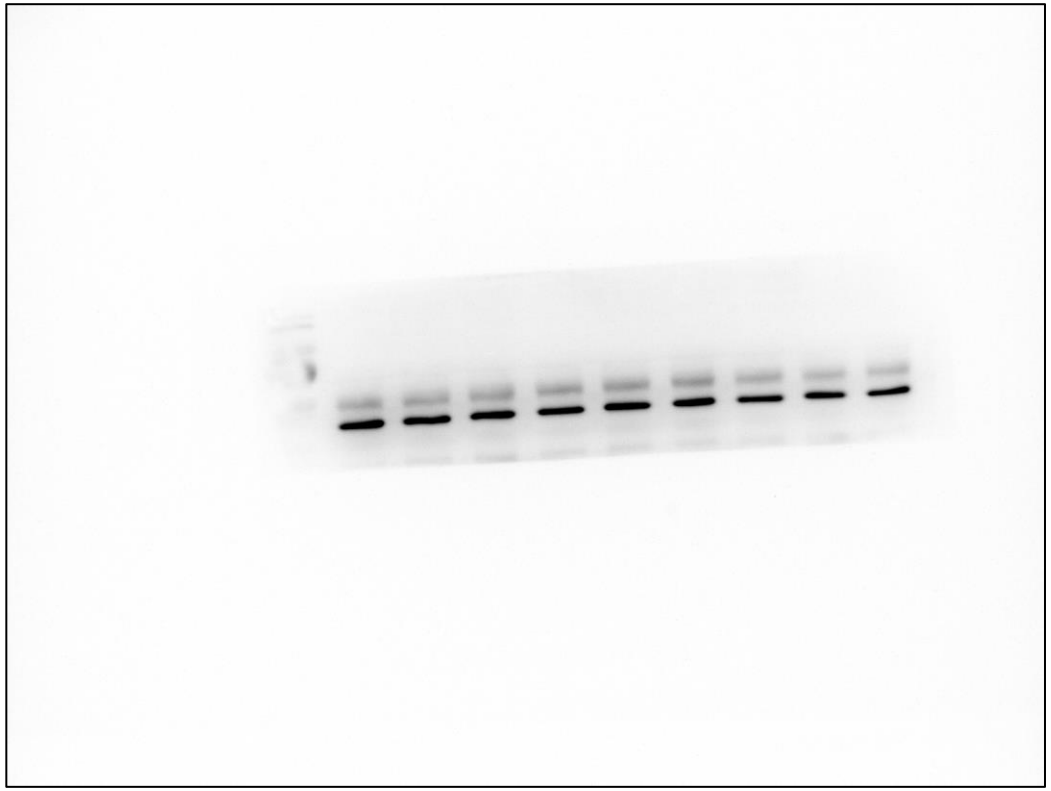

WB_Fig3_RIP3_actin

Figure 3

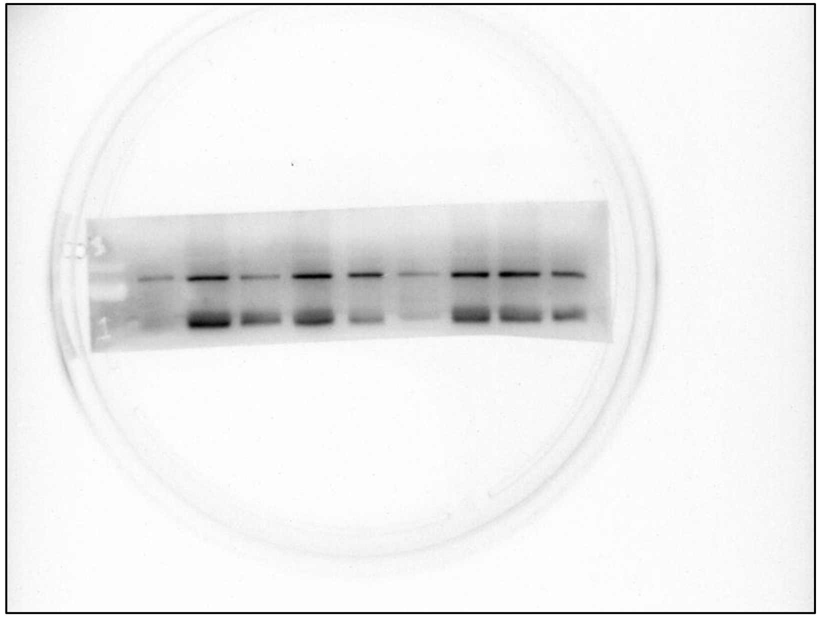

WB_Fig3_MLKL

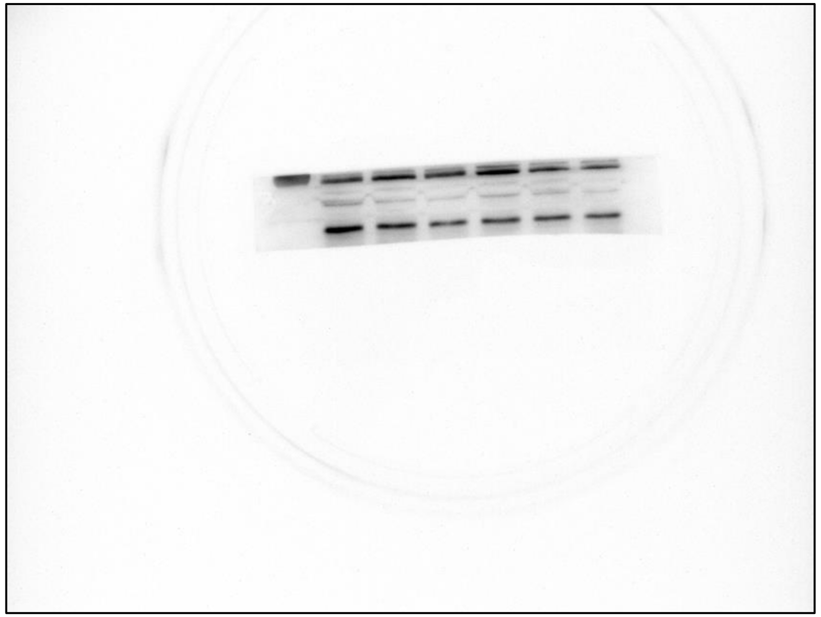

WB_Fig3_pMLKL

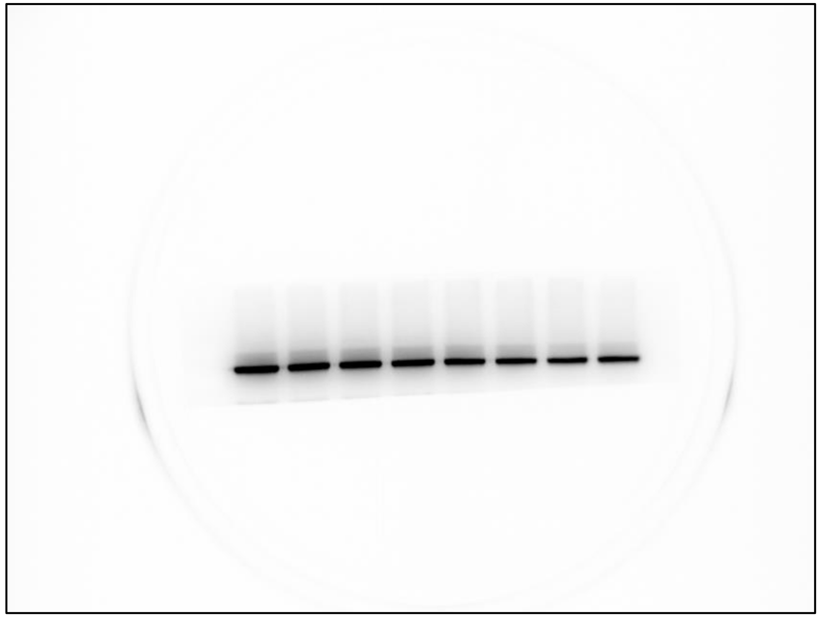

WB_Fig3_(p)MLKL_actin

Figure 6

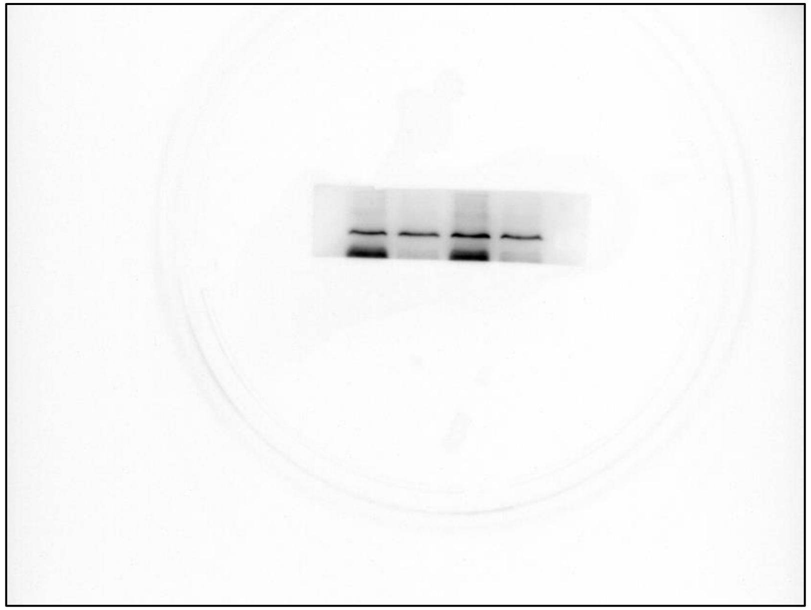

WB_Fig6_RIP1

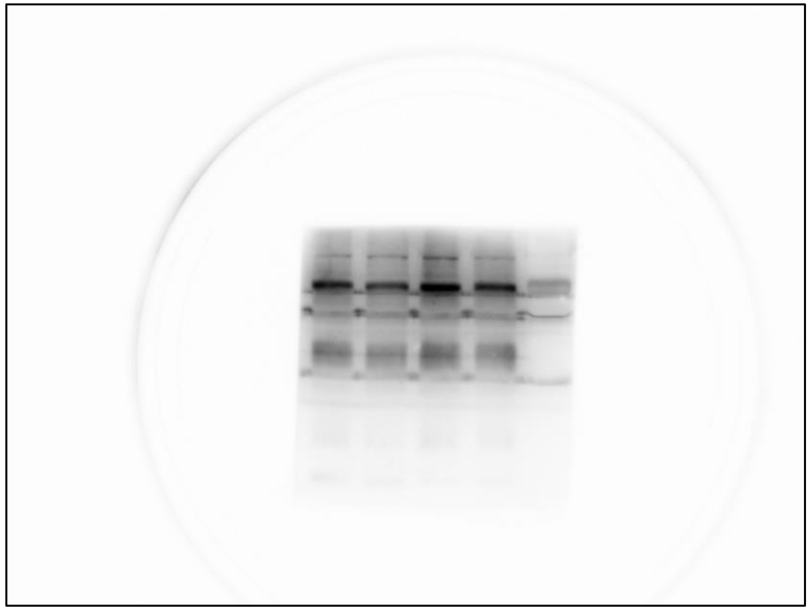

WB_Fig6_RIP3

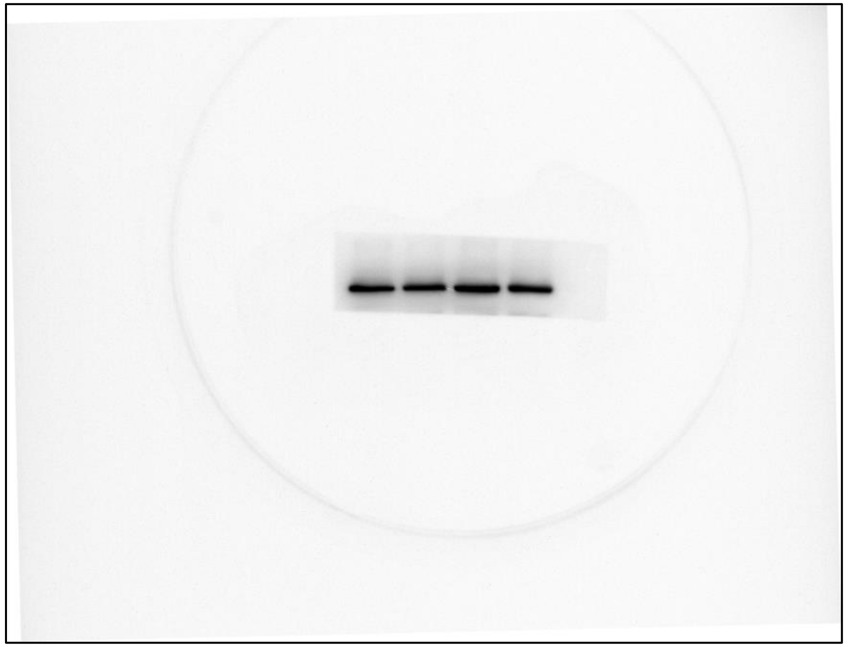

WB_Fig6_RIP1,3_actin

Figure 6

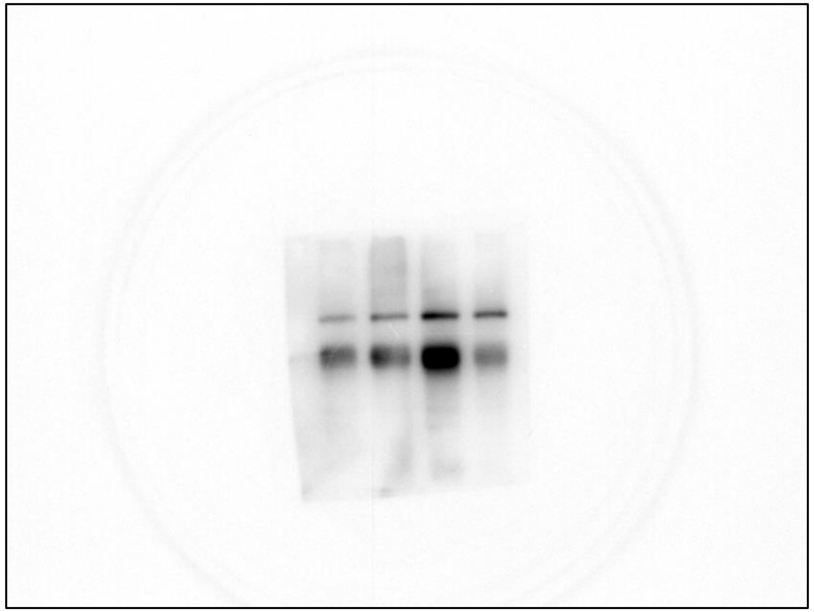

WB_Fig6_MLKL

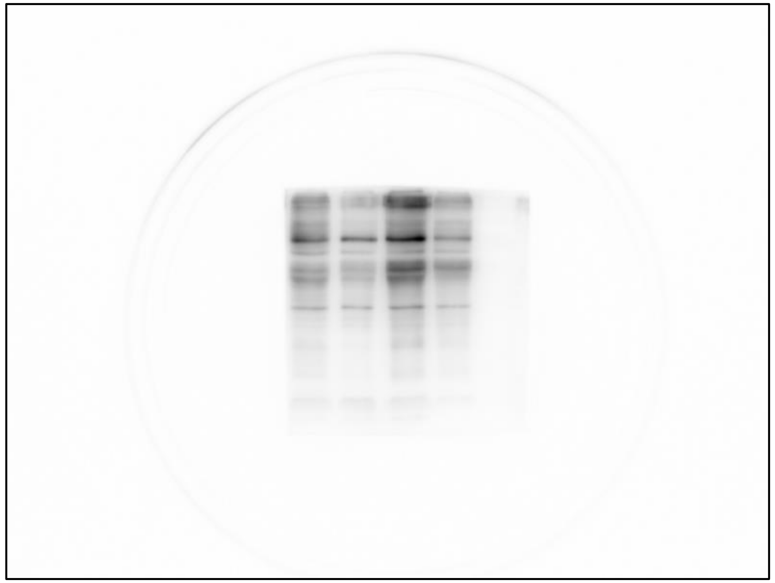

WB_Fig6_pMLKL

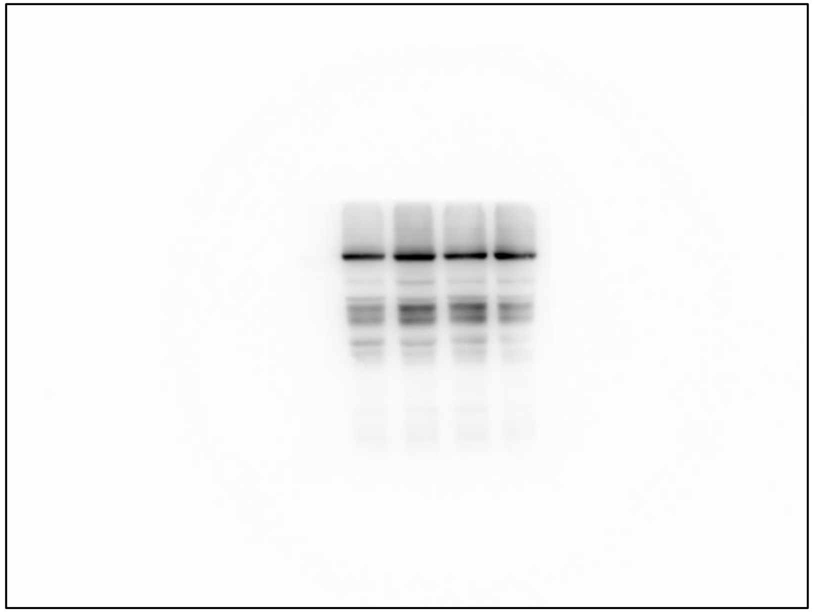

WB_Fig6_(p)MLKL_actin
