# Supplementary material for: RIP3-mediated microglial necroptosis promotes neuroinflammation and neurodegeneration in the early stages of diabetic retinopathy
Source: Cell Death Dis. 2023 Mar 29;14(3):227. doi: 10.1038/s41419-023-05660-z (PMC10060420; doi:10.1038/s41419-023-05660-z)
Supplement: Supplementary file 1 — Co-author approves for adding a new author [file 41419_2023_5660_MOESM1_ESM.pdf]

回复：Request for adding an author in our manuscript CDDIS-22-3496

发件人： **眼科研究室-梁嘉健** <ljj@jsiec.org>

收件人： 医生-黄梓敬 <huangzj@jsiec.org>

时 间： 2023年2月3日(星期五) 18:02

Dear Dr. Huang,
Thank you very much for the e-mail and I totally agree to add Dr. Brelen as a co-author due to his hard work for the paper.

Best wishes,
Jiajian

发件人：医生-黄梓敬 <huangzj@jsiec.org>
发送时间：2023年2月2日(星期四) 21:31
收件人：医生-陈伟奇 <cwq@jsiec.org>; 刘庆平 <qingpingliu40@126.com>; Michael Tsz Kin NG <micntk@hotmail.com>; 医生-黄定国 <hdg@jsiec.org>; 眼科研究室-梁嘉健 <ljj@jsiec.org>; 眼科研究室-陈少琅 <csl@jsiec.org>; 研究生-杨入翠 <yrc@jsiec.org>; 研究生-谢碧瑶 <xby@jsiec.org>; 研究生-柯舒萍 <ksp@jsiec.org>
抄 送：Marten Brelen (OVS) <marten.brelen@cuhk.edu.hk>
主 题：Request for adding an author in our manuscript CDDIS-22-3496

Dear co-authors,

Thank you very much for your contribution to our manuscript entitled "RIP3-mediated microglial necroptosis promotes neuroinflammation and neurodegeneration in the early stages of diabetic retinopathy" (ID: CDDIS-22-3496). The manuscript has been provisionally accepted for publication in Cell Death & Disease.

I am writing to you because I am wishing to add Dr. Marten E. Brelén as an extra author, who has contributed much to the revised version of our manuscript. After we received the referee's comments, he did a great job for the review of the whole manuscript for grammatical accuracy and clarity. We could not finish the revised version without his hard work. Therefore, I wish to add Dr. Brelén as a new author to the paper. Please find attached the title page of the manuscript.

If you agree to this proposal, please kindly respond to this email. Please also contact me if you have disagreement or any other question.

Thank you again for your kind contribution to this work.

Best wishes,

Zijing Huang

发件人：cddisease <cddisease@springernature.com>
发送时间：2023年2月2日(星期四) 00:45
收件人：医生-黄梓敬 <huangzj@jsiec.org>
主 题：CDDIS-22-3496RR Initial Quality Check

Dear Dr Huang,

In checking in your manuscript submitted to Cell Death & Disease it has come to our attention that the following must be addressed before we can proceed.

1. It has come to our attention that your most recent author list differs from the one in your original submission. We find that the following authors have been added since your initial submission: Marten E. Brelén.

Please request agreement from all authors including additions and deletions, these can be collected in the following way:

Email your co-authors with the change, and ask them to reply to your email confirming that they agree to these changes. Once you have collected these replies, please combine all of the co-authors' email responses in one document and upload this file to your submission

2. Please make sure you include a data availability statement in your Article file under its own subheading.

回复：Request for adding an author in our manuscript CDDIS-22-3496

发件人： **眼科研究室-陈少琅** <csl@jsiec.org>

收件人： 医生-黄梓敬 <huangzj@jsiec.org>

时 间： 2023年2月2日(星期四) 22:07

Dear Dr Huang,

Thank you for the e-mail and I totally agree to add Dr. Marten E. Brelén as a co-author due to his work for the paper.

Best,

Shaolang Chen

发件人：医生-黄梓敬 <huangzj@jsiec.org>

发送时间：2023年2月2日(星期四) 21:31

收件人：医生-陈伟奇 <cwq@jsiec.org>; 刘庆平 <qingpingliu40@126.com>; Michael Tsz Kin NG <micntk@hotmail.com>; 医生-黄定国 <hdg@jsiec.org>; 眼科研究室-梁嘉健 <ljj@jsiec.org>; 眼科研究室-陈少琅 <csl@jsiec.org>; 研究生-杨入翠 <yrc@jsiec.org>; 研究生-谢碧瑶 <xby@jsiec.org>; 研究生-柯舒萍 <ksp@jsiec.org>

抄 送：Marten Brelen (OVS) <marten.brelen@cuhk.edu.hk>

主 题：Request for adding an author in our manuscript CDDIS-22-3496

Dear co-authors,

Thank you very much for your contribution to our manuscript entitled "RIP3-mediated microglial necroptosis promotes neuroinflammation and neurodegeneration in the early stages of diabetic retinopathy" (ID: CDDIS-22-3496). The manuscript has been provisionally accepted for publication in Cell Death & Disease.

I am writing to you because I am wishing to add Dr. Marten E. Brelén as an extra author, who has contributed much to the revised version of our manuscript. After we received the referee's comments, he did a great job for the review of the whole manuscript for grammatical accuracy and clarity. We could not finish the revised version without his hard work. Therefore, I wish to add Dr. Brelén as a new author to the paper. Please find attached the title page of the manuscript.

If you agree to this proposal, please kindly respond to this email. Please also contact me if you have disagreement or any other question.

Thank you again for your kind contribution to this work.

Best wishes,

Zijing Huang

发件人：cddisease <cddisease@springernature.com>

发送时间：2023年2月2日(星期四) 00:45

收件人：医生-黄梓敬 <huangzj@jsiec.org>

主 题：CDDIS-22-3496RR Initial Quality Check

Dear Dr Huang,

In checking in your manuscript submitted to Cell Death & Disease it has come to our attention that the following must be addressed before we can proceed.

1. It has come to our attention that your most recent author list differs from the one in your original submission. We find that the following authors have been added since your initial submission: Marten E. Brelén.

Please request agreement from all authors including additions and deletions, these can be collected in the following way:

Email your co-authors with the change, and ask them to reply to your email confirming that they agree to these changes. Once you have collected these replies, please combine all of the co-authors' email responses in one document and upload this file to your submission

2. Please make sure you include a data availability statement in your Article file under its own subheading.

Re: Request for adding an author in our manuscript CDDIS-22-3496

发件人： **Michael Tsz Kin NG** <micntk@hotmail.com>
收件人： 医生-黄梓敬 <huangzj@jsiec.org>
时 间： 2023年2月3日(星期五) 17:27

Approve. Thanks!

From: 梓敬 <huangzj@jsiec.org>
Sent: 02 February 2023 21:31
To: 医生-陈伟奇 <cwq@jsiec.org>; 刘庆平 <qingpingliu40@126.com>; Michael Tsz Kin NG <micntk@hotmail.com>; 医生-黄定国 <hdg@jsiec.org>; 眼科研究室-梁嘉健 <ljj@jsiec.org>; 眼科研究室-陈少琅 <csl@jsiec.org>; 研究生-杨入翠 <yrc@jsiec.org>; 研究生-谢碧瑶 <xby@jsiec.org>; 研究生-柯舒萍 <ksp@jsiec.org>
Cc: Marten Brelen (OVS) <marten.brelen@cuhk.edu.hk>
Subject: Request for adding an author in our manuscript CDDIS-22-3496

Dear co-authors,

Thank you very much for your contribution to our manuscript entitled "RIP3-mediated microglial necroptosis promotes neuroinflammation and neurodegeneration in the early stages of diabetic retinopathy" (ID: CDDIS-22-3496). The manuscript has been provisionally accepted for publication in Cell Death & Disease.

I am writing to you because I am wishing to add Dr. Marten E. Brelén as an extra author, who has contributed much to the revised version of our manuscript. After we received the referee's comments, he did a great job for the review of the whole manuscript for grammatical accuracy and clarity. We could not finish the revised version without his hard work. Therefore, I wish to add Dr. Brelén as a new author to the paper. Please find attached the title page of the manuscript.

If you agree to this proposal, please kindly respond to this email. Please also contact me if you have disagreement or any other question.

Thank you again for your kind contribution to this work.

Best wishes,

Zijing Huang

发件人：cddisease <cddisease@springernature.com>
发送时间：2023年2月2日(星期四) 00:45
收件人：医生-黄梓敬 <huangzj@jsiec.org>
主 题：CDDIS-22-3496RR Initial Quality Check

Dear Dr Huang,

In checking in your manuscript submitted to Cell Death & Disease it has come to our attention that the following must be addressed before we can proceed.

1. It has come to our attention that your most recent author list differs from the one in your original submission. We find that the following authors have been added since your initial submission: Marten E. Brelén.

Please request agreement from all authors including additions and deletions, these can be collected in the following way:

Email your co-authors with the change, and ask them to reply to your email confirming that they agree to these changes. Once you have collected these replies, please combine all of the co-authors' email responses in one document and upload this file to your submission

2. Please make sure you include a data availability statement in your Article file under its own subheading.

3. All Articles must be accompanied by a Reproducibility checklist (http://www.nature.com/authors/policies/checklist_AJ.pdf) ensuring you do not leave any of the boxes blank, please write 'n/a' instead if they do not apply.

Re:Request for adding an author in our manuscript CDDIS-22-3496

发件人： 刘 <qingpingliu40@126.com>

收件人： 医生-黄梓敬 <huangzj@jsiec.org>

时 间： 2023年2月3日(星期五) 11:07

Dear Zijing

Based on Dr. Marten E. Brelen's contributions to this article, I agree to add Dr. Marten E. Brelen as the new author of this article.

Best

Qingping Liu

--

刘庆平
生命科学与技术学院
辽宁省糖脂代谢研究重点实验室
大连大学
大连经济技术开发区学府大街10号 116622

Qingping Liu Ph.D M.D

Prof

qingpingliu40@126.com

College of Life Science and Technology ,

Dalian University,

10Xue Fu Avenue,Economic&Techincal Development Zone,

Dalian 116622,

China

Tel: 86-41187402134

Fax: 86-41187402134

At 2023-02-02 21:31:53, "梓敬" <huangzj@jsiec.org> wrote:

Dear co-authors,

Thank you very much for your contribution to our manuscript entitled “RIP3-mediated microglial necroptosis promotes neuroinflammation and neurodegeneration in the early stages of diabetic retinopathy” (ID: CDDIS-22-3496). The manuscript has been provisionally accepted for publication in Cell Death & Disease.

I am writing to you because I am wishing to add Dr. Marten E. Brelén as an extra author, who has contributed much to the revised version of our manuscript. After we received the referee's comments, he did a great job for the review of the whole manuscript for grammatical accuracy and clarity. We could not finish the revised version without his hard work. Therefore, I wish to add Dr. Brelén as a new author to the paper. Please find attached the title page of the manuscript.

If you agree to this proposal, please kindly respond to this email. Please also contact me if you have disagreement or any other question.

Thank you again for your kind contribution to this work.

Best wishes,

Zijing Huang

回复：Request for adding an author in our manuscript CDDIS-22-3496

发件人： **研究生-杨入翠** <yrc@jsiec.org>
收件人： 医生-黄梓敬 <huangzj@jsiec.org>
时　间： 2023年2月2日(星期四) 21:58

Dear Dr. Zijing Huang,

Thank you for your letter. Since Dr. Marten E. Brelén has made great contributions to the revision of the article, I strongly agree to include him as one of the coauthors in this paper(ID: CDDIS-22-3496).

Kind regards,
Ms.Yang Rucui

发件人：医生-黄梓敬 <huangzj@jsiec.org>
发送时间：2023年2月2日(星期四) 21:31
收件人：医生-陈伟奇 <cwq@jsiec.org>; 刘庆平 <qingpingliu40@126.com>; Michael Tsz Kin NG <micntk@hotmail.com>; 医生-黄定国 <hdg@jsiec.org>; 眼科研究室-梁嘉健 <ljj@jsiec.org>; 眼科研究室-陈少琅 <csl@jsiec.org>; 研究生-杨入翠 <yrc@jsiec.org>; 研究生-谢碧瑶 <xby@jsiec.org>; 研究生-柯舒萍 <ksp@jsiec.org>
抄　送：Marten Brelen (OVS) <marten.brelen@cuhk.edu.hk>
主　题：Request for adding an author in our manuscript CDDIS-22-3496

Dear co-authors,

Thank you very much for your contribution to our manuscript entitled “RIP3-mediated microglial necroptosis promotes neuroinflammation and neurodegeneration in the early stages of diabetic retinopathy” (ID: CDDIS-22-3496). The manuscript has been provisionally accepted for publication in Cell Death & Disease.

I am writing to you because I am wishing to add Dr. Marten E. Brelén as an extra author, who has contributed much to the revised version of our manuscript. After we received the referee's comments, he did a great job for the review of the whole manuscript for grammatical accuracy and clarity. We could not finish the revised version without his hard work. Therefore, I wish to add Dr. Brelén as a new author to the paper. Please find attached the title page of the manuscript.

If you agree to this proposal, please kindly respond to this email. Please also contact me if you have disagreement or any other question.

Thank you again for your kind contribution to this work.

Best wishes,

Zijing Huang

发件人：cddisease <cddisease@springernature.com>
发送时间：2023年2月2日(星期四) 00:45
收件人：医生-黄梓敬 <huangzj@jsiec.org>
主　题：CDDIS-22-3496RR Initial Quality Check

Dear Dr Huang,

In checking in your manuscript submitted to Cell Death & Disease it has come to our attention that the following must be addressed before we can proceed.

1. It has come to our attention that your most recent author list differs from the one in your original submission. We find that the following authors have been added since your initial submission: Marten E. Brelén.

Please request agreement from all authors including additions and deletions, these can be collected in the following way:

Email your co-authors with the change, and ask them to reply to your email confirming that they agree to these changes. Once you have

Re:Request for adding an author in our manuscript CDDIS-22-3496

发件人： **研究生-谢碧瑶** <xby@jsiec.org>

收件人： 医生-黄梓敬 <huangzj@jsiec.org>

时 间： 2023年2月2日(星期四) 21:45

Dear Dr Huang

Email received, the changes are fine with me. Thank you.

Best wishes

Biyao Xie

from 阿里邮箱 iPhone

发件人：梓敬<huangzj@jsiec.org>
日 期：2023年02月02日 21:31:53
收件人：医生-陈伟奇<cwq@jsiec.org>; 刘庆平<qingpingliu40@126.com>; Michael Tsz Kin NG<micntk@hotmail.com>; 医生-黄定国<hdg@jsiec.org>; 眼科研究室-梁嘉健<ljj@jsiec.org>; 眼科研究室-陈少琅<csl@jsiec.org>; 研究生-杨入翠<yrc@jsiec.org>; 研究生-谢碧瑶<xby@jsiec.org>; 研究生-柯舒萍<ksp@jsiec.org>
抄 送：Marten Brelen (OVS)<marten.brelen@cuhk.edu.hk>
主 题：Request for adding an author in our manuscript CDDIS-22-3496

Dear co-authors,

Thank you very much for your contribution to our manuscript entitled "RIP3-mediated microglial necroptosis promotes neuroinflammation and neurodegeneration in the early stages of diabetic retinopathy" (ID: CDDIS-22-3496). The manuscript has been provisionally accepted for publication in Cell Death & Disease.

I am writing to you because I am wishing to add Dr. Marten E. Brelén as an extra author, who has contributed much to the revised version of our manuscript. After we received the referee's comments, he did a great job for the review of the whole manuscript for grammatical accuracy and clarity. We could not finish the revised version without his hard work. Therefore, I wish to add Dr. Brelén as a new author to the paper. Please find attached the title page of the manuscript.

If you agree to this proposal, please kindly respond to this email. Please also contact me if you have disagreement or any other question.

Thank you again for your kind contribution to this work.

Best wishes,

Zijing Huang

发件人：cddisease <cddisease@springernature.com>
发送时间：2023年2月2日(星期四) 00:45
收件人：医生-黄梓敬 <huangzj@jsiec.org>
主 题：CDDIS-22-3496RR Initial Quality Check

Dear Dr Huang,

In checking in your manuscript submitted to Cell Death & Disease it has come to our attention that the following must be addressed before we can proceed.

1. It has come to our attention that your most recent author list differs from the one in your original submission. We find that the following authors have been added since your initial submission: Marten E. Brelén.

Please request agreement from all authors including additions and deletions, these can be collected in the following way:

Email your co-authors with the change, and ask them to reply to your email confirming that they agree to these changes. Once you have collected these replies, please combine all of the co-authors' email responses in one document and upload this file to your submission

2. Please make sure you include a data availability statement in your Article file under its own subheading.

回复：Request for adding an author in our manuscript CDDIS-22-3496

发件人： 研究生-柯舒萍 <ksp@jsiec.org>

收件人： 医生-黄梓敬 <huangzj@jsiec.org>

时 间： 2023年2月3日(星期五) 18:34

Dear Dr Huang
Email received, the changes are fine with me. Thank you.
Best wishes
Shuping Ke

发件人：梓敬<huangzj@jsiec.org>
日 期：2023年02月02日 21:31:53
收件人：医生-陈伟奇<cwq@jsiec.org>; 刘庆平<qingpingliu40@126.com>; Michael Tsz Kin NG<micntk@hotmail.com>; 医生-黄定国<hdg@jsiec.org>; 眼科研究室-梁嘉健<ljj@jsiec.org>; 眼科研究室-陈少琅<csl@jsiec.org>; 研究生-杨入翠<yrc@jsiec.org>; 研究生-谢碧瑶<xby@jsiec.org>; 研究生-柯舒萍<ksp@jsiec.org>
抄 送：Marten Brelen (OVS)<marten.brelen@cuhk.edu.hk>
主 题：Request for adding an author in our manuscript CDDIS-22-3496

Dear co-authors,

Thank you very much for your contribution to our manuscript entitled "RIP3-mediated microglial necroptosis promotes neuroinflammation and neurodegeneration in the early stages of diabetic retinopathy" (ID: CDDIS-22-3496). The manuscript has been provisionally accepted for publication in Cell Death & Disease.

I am writing to you because I am wishing to add Dr. Marten E. Brelén as an extra author, who has contributed much to the revised version of our manuscript. After we received the referee's comments, he did a great job for the review of the whole manuscript for grammatical accuracy and clarity. We could not finish the revised version without his hard work. Therefore, I wish to add Dr. Brelén as a new author to the paper. Please find attached the title page of the manuscript.

If you agree to this proposal, please kindly respond to this email. Please also contact me if you have disagreement or any other question.

Thank you again for your kind contribution to this work.

Best wishes,

Zijing Huang

发件人：cddisease <cddisease@springernature.com>
发送时间：2023年2月2日(星期四) 00:45
收件人：医生-黄梓敬 <huangzj@jsiec.org>
主 题：CDDIS-22-3496RR Initial Quality Check

Dear Dr Huang,

In checking in your manuscript submitted to Cell Death & Disease it has come to our attention that the following must be addressed before we can proceed.

1. It has come to our attention that your most recent author list differs from the one in your original submission. We find that the following authors have been added since your initial submission: Marten E. Brelén.

Please request agreement from all authors including additions and deletions, these can be collected in the following way:

Email your co-authors with the change, and ask them to reply to your email confirming that they agree to these changes. Once you have collected these replies, please combine all of the co-authors' email responses in one document and upload this file to your submission

2. Please make sure you include a data availability statement in your Article file under its own subheading.

3. All Articles must be accompanied by a Reproducibility checklist (http://www.nature.com/authors/policies/checklist_AJ.pdf) ensuring you do not leave any of the boxes blank, please write 'n/a' instead if they do not apply.

Re: Request for adding an author in our manuscript CDDIS-22-3496

发件人： **医生-陈伟奇** <cwq@jsiec.org>

收件人： 医生-黄梓敬 <huangzj@jsiec.org> ；刘庆平 <qingpingliu40@126.com> ；Michael Tsz Kin NG <micntk@hotmail.com> ；医生-黄定国 <hdg@jsiec.org> ；眼科研究室-梁嘉健 <ljj@jsiec.org> ；眼科研究室-陈少琅 <csl@jsiec.org> ；研究生-杨入翠 <yrc@jsiec.org> ；研究生-谢碧瑶 <xby@jsiec.org> ；研究生-柯舒萍 <ksp@jsiec.org>

抄 送： Marten Brelen (OVS) <marten.brelen@cuhk.edu.hk>

时 间： 2023年2月2日(星期四) 21:50

Dear Zijing,
Thank you for the e-mail and I totally agree to add Dr. Brelen as a co-author due to his hard work for the paper.
Best,
Weiqi Chen

cwq@jsiec.org

From: 梓敬
Date: 2023-02-02 21:31
To: 医生-陈伟奇; 刘庆平; Michael Tsz Kin NG; 医生-黄定国; 眼科研究室-梁嘉健; 眼科研究室-陈少琅; 研究生-杨入翠; 研究生-谢碧瑶; 研究生-柯舒萍
CC: Marten Brelen (OVS)
Subject: Request for adding an author in our manuscript CDDIS-22-3496

Dear co-authors,

Thank you very much for your contribution to our manuscript entitled "RIP3-mediated microglial necroptosis promotes neuroinflammation and neurodegeneration in the early stages of diabetic retinopathy" (ID: CDDIS-22-3496). The manuscript has been provisionally accepted for publication in Cell Death & Disease.

I am writing to you because I am wishing to add Dr. Marten E. Brelén as an extra author, who has contributed much to the revised version of our manuscript. After we received the referee's comments, he did a great job for the review of the whole manuscript for grammatical accuracy and clarity. We could not finish the revised version without his hard work. Therefore, I wish to add Dr. Brelén as a new author to the paper. Please find attached the title page of the manuscript.

If you agree to this proposal, please kindly respond to this email. Please also contact me if you have disagreement or any other question.

Thank you again for your kind contribution to this work.

Best wishes,

Zijing Huang

发件人：cddisease <cddisease@springernature.com>
发送时间：2023年2月2日(星期四) 00:45
收件人：医生-黄梓敬 <huangzj@jsiec.org>
主 题：CDDIS-22-3496RR Initial Quality Check

Dear Dr Huang,

In checking in your manuscript submitted to Cell Death & Disease it has come to our attention that the following must be addressed before we can proceed.

1. It has come to our attention that your most recent author list differs from the one in your original submission. We find that the following authors have been added since your initial submission: Marten E. Brelén.

Please request agreement from all authors including additions and deletions, these can be collected in the following way:

Email your co-authors with the change, and ask them to reply to your email confirming that they agree to these changes. Once you have collected these replies, please combine all of the co-authors' email responses in one document and upload this file to your submission

2. Please make sure you include a data availability statement in your Article file under its own subheading.

回复：Request for adding an author in our manuscript CDDIS-22-3496

发件人： **医生-黄定国** <hdg@jsiec.org>
收件人： 医生-黄梓敬 <huangzj@jsiec.org>
时 间： 2023年2月2日(星期四) 21:40

Dear Dr Huang：

I agree to the changes made by the corresponding author. Thank you very much.

Best wishes,

Dingguo Huang

发件人：医生-黄梓敬 <huangzj@jsiec.org>
发送时间：2023年2月2日(星期四) 21:31
收件人：医生-陈伟奇 <cwq@jsiec.org>; 刘庆平 <qingpingliu40@126.com>; Michael Tsz Kin NG <micntk@hotmail.com>; 医生-黄定国 <hdg@jsiec.org>; 眼科研究室-梁嘉健 <ljj@jsiec.org>; 眼科研究室-陈少琅 <csl@jsiec.org>; 研究生-杨入翠 <yrc@jsiec.org>; 研究生-谢碧瑶 <xby@jsiec.org>; 研究生-柯舒萍 <ksp@jsiec.org>
抄 送：Marten Brelen (OVS) <marten.brelen@cuhk.edu.hk>
主 题：Request for adding an author in our manuscript CDDIS-22-3496

Dear co-authors,

Thank you very much for your contribution to our manuscript entitled "RIP3-mediated microglial necroptosis promotes neuroinflammation and neurodegeneration in the early stages of diabetic retinopathy" (ID: CDDIS-22-3496). The manuscript has been provisionally accepted for publication in Cell Death & Disease.

I am writing to you because I am wishing to add Dr. Marten E. Brelén as an extra author, who has contributed much to the revised version of our manuscript. After we received the referee's comments, he did a great job for the review of the whole manuscript for grammatical accuracy and clarity. We could not finish the revised version without his hard work. Therefore, I wish to add Dr. Brelén as a new author to the paper. Please find attached the title page of the manuscript.

If you agree to this proposal, please kindly respond to this email. Please also contact me if you have disagreement or any other question.

Thank you again for your kind contribution to this work.

Best wishes,

Zijing Huang

发件人：cddisease <cddisease@springernature.com>
发送时间：2023年2月2日(星期四) 00:45
收件人：医生-黄梓敬 <huangzj@jsiec.org>
主 题：CDDIS-22-3496RR Initial Quality Check

Dear Dr Huang,

In checking in your manuscript submitted to Cell Death & Disease it has come to our attention that the following must be addressed before we can proceed.

1. It has come to our attention that your most recent author list differs from the one in your original submission. We find that the following authors have been added since your initial submission: Marten E. Brelén.

Please request agreement from all authors including additions and deletions, these can be collected in the following way:

Email your co-authors with the change, and ask them to reply to your email confirming that they agree to these changes. Once you have
